# Recent Advances in Metabolic Engineering for the Biosynthesis of Phosphoenol Pyruvate–Oxaloacetate–Pyruvate-Derived Amino Acids

**DOI:** 10.3390/molecules29122893

**Published:** 2024-06-18

**Authors:** Lianghong Yin, Yanan Zhou, Nana Ding, Yu Fang

**Affiliations:** 1 State Key Laboratory of Subtropical Silviculture, Zhejiang A&F University, Hangzhou 311300, China; ylh4@163.com (L.Y.); zhouyanan202101@163.com (Y.Z.); 2 Zhejiang Provincial Key Laboratory of Resources Protection and Innovation of Traditional Chinese Medicine, Zhejiang A&F University, Hangzhou 311300, China

**Keywords:** metabolic engineering, *Escherichia coli*, *Corynebacterium glutamicum*, phosphoenol–pyruvate–oxaloacetate–pyruvate node, amino acids

## Abstract

The phosphoenol pyruvate–oxaloacetate–pyruvate-derived amino acids (POP-AAs) comprise native intermediates in cellular metabolism, within which the phosphoenol pyruvate–oxaloacetate–pyruvate (POP) node is the switch point among the major metabolic pathways existing in most living organisms. POP-AAs have widespread applications in the nutrition, food, and pharmaceutical industries. These amino acids have been predominantly produced in *Escherichia coli* and *Corynebacterium glutamicum* through microbial fermentation. With the rapid increase in market requirements, along with the global food shortage situation, the industrial production capacity of these two bacteria has encountered two bottlenecks: low product conversion efficiency and high cost of raw materials. Aiming to push forward the update and upgrade of engineered strains with higher yield and productivity, this paper presents a comprehensive summarization of the fundamental strategy of metabolic engineering techniques around phosphoenol pyruvate–oxaloacetate–pyruvate node for POP-AA production, including L-tryptophan, L-tyrosine, L-phenylalanine, L-valine, L-lysine, L-threonine, and L-isoleucine. Novel heterologous routes and regulation methods regarding the carbon flux redistribution in the POP node and the formation of amino acids should be taken into consideration to improve POP-AA production to approach maximum theoretical values. Furthermore, an outlook for future strategies of low-cost feedstock and energy utilization for developing amino acid overproducers is proposed.

## 1. Introduction

Amino acids comprise small molecular chemicals harboring variable groups such as indole, hydroxyl, phenyl, methyl, carboxy, and amidogen groups. These functional groups promote the utility of amino acids as building block compounds, which enjoy considerable market demand and are previously produced through plant or animal extraction [1]. Because certain plants or animals represent an expensive resource and their extraction generates acid waste and affords negative environmental impact, it is recommended that plant- or animal-based extract production be replaced by biotechnology-based production from renewable carbon resources [2]. For instance, the utilization of microorganisms enables the production of various polyhydroxyalkanoates (PHAs) from sugars derived from lignocellulosic biomass (LCB), hydrolysates, or aromatic compounds, offering a potential solution to tackle environmental challenges [3]. Furthermore, researchers are focusing on novel substrates, strain enhancements, minimizing by-products, enhancing product tolerance, and optimizing fermentation conditions to boost bioethanol yield. This eco-friendly and renewable alternative aims to replace fossil-based fuels [4]. Additionally, aiming to rival chemical or enzymatic methods in amino acid production, advancements in systems and synthetic biology have sparked novel technologies, paving a promising path for engineering amino acid-producing microorganisms [5,6]. In amino acid production via fermentation, two bacterial species stand out as the most frequently utilized: *Escherichia coli* (*E. coli*) and *Corynebacterium glutamicum* (*C. glutamicum*) [7]. Both of these bacteria have demonstrated their proficiency in synthesizing a diverse range of amino acids, and researchers have implemented various metabolic engineering modifications to enhance their efficiency as amino acid producers [7].

**Figure 1:**
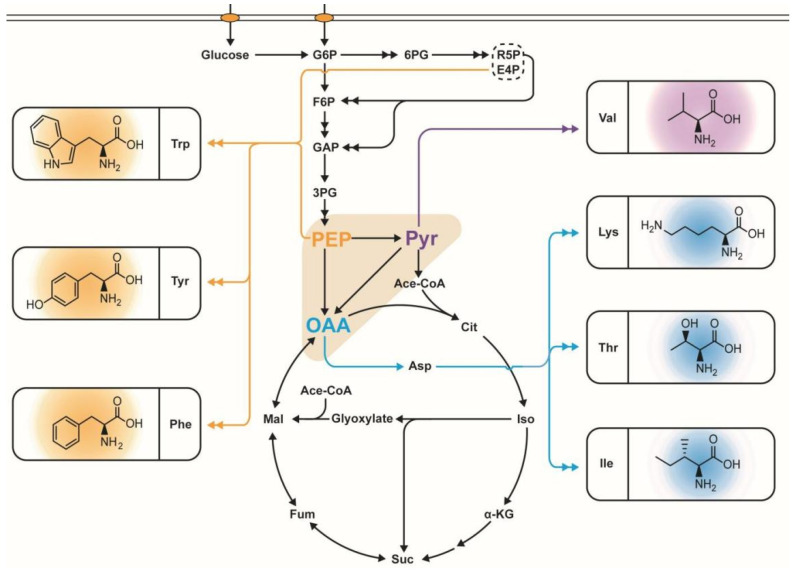
Metabolic pathways of amino acids. 3-PG, glycerate-3-phosphate; E4P, erythrose-4-phosphate; G6P, glucose-6-phosphate; Glc, glucose; L-Asp, aspartate; OAA, oxaloacetate; PEP, phosphoenolpyruvate; Pyr, pyruvate.

Most amino acids constitute metabolites or products of branched generation routes that are natively synthesized in microbes among these branched routes [8]; the use of phosphoenolpyruvate (PEP), pyruvate (PYR), and oxaloacetate (OAA) as starting materials for the biosynthesis of corresponding amino acids is critical. Chemical compounds in the branched metabolic pathway that are derived from the phosphoenol pyruvate–oxaloacetate–pyruvate (POP) node include L-tryptophan (L-Trp), L-tyrosine (L-Tyr), L-phenylalanine (L-Phe), L-valine (L-Val) L-lysine (L-Lys), L-threonine (L-Thr), and L-isoleucine (L-Ile). The enzymes at the POP node in bacteria direct the carbon flux to corresponding amino acid biosynthesis (Figure 1).

Three enzymes catalyze the C3-carboxylation and C4-decarboxylation reactions; these enzymes are PEP carboxylase, PEP carboxykinase, and malic enzyme, which are common enzymes shared by *E. coli* and *C. glutamicum.* However, *C. glutamicum* also possesses additional PYR carboxylase and OAA decarboxylase for PYR carboxylation and OAA decarboxylation. In *E. coli*, PEP is converted to OAA by PEP carboxylase. Malic enzymes convert malate to PYR via two isoenzymes encoded by *maeB* and *sfcA* with cofactor specificities for NADP and NAD, respectively. Then, PYR undergoes gluconeogenesis to form PEP by PEP synthetase. Alternatively, PEP is also synthesized through a decarboxylation reaction from OAA by PEP carboxykinase. In *C. glutamicum*, PEP carboxylase and PYR carboxylase convert PEP and PYR to OAA, in which PYR carboxylase contributes about 90% of the total OAA formation. OAA and malate are decarboxylated to PEP and PYR via PEP carboxykinase, OAA decarboxylase, and malic enzyme. PEP serves as a phosphoryl donor for glucose uptake via the phosphotransferase system in *E. coli* and *C. glutamicum*, which is the material for the PYR kinase-catalyzed anaplerotic reaction at the same time. Then, the PYR is oxidatively decarboxylated to acetyl-CoA by the PYR dehydrogenase complex. In addition, accumulated PYR is converted to acetate by PYR: quinone oxidoreductase. Then, acetate is converted to acetyl-CoA by acetate kinase and phosphotransacetylase. Similarly, accumulated PYR is catalyzed to L-alanine and lactate via alanine aminotransferase and lactate dehydrogenase, respectively. Metabolic regulation of POP nodes is very complicated. Along with aspartate and malate inhibiting PEP carboxylase in *E. coli* and *C. glutamicum* via feedback mechanisms, and acetyl-CoA and fructose-1,6-P activating it [9], acetyl-CoA and oxaloacetate adversely inhibit the activity of *E. coli* malic enzyme MaeB [10]. ATP inhibits SfcA, and aspartate activates it, while oxaloacetate and glutamate inhibit and NH4^+^ activates the *C. glutamicum* malic enzyme MalE [11]. *E. coli* PEP carboxykinase and synthetase are subject to feedback inhibition by PEP. In addition, *C. glutamicum* PYR carboxylase and PEP carboxykinase are inhibited by aspartate, acetyl-CoA, ADP, AMP, and ATP [12]. Oxaloacetate decarboxylase is subject to feedback inhibition by succinate and CoA. PYR kinase (PYK) is inhibited by ATP and GTP and induced by AMP. The expression of *pckA*, *ppsA*, *maeB*, and *sfcA* is repressed by glucose. In addition, *pckA* is subjected to inhibition from the global transcriptional regulator Crp. Moreover, FruR is a catabolite repressor/activator, which can activate *ppsA* and *pckA* while repressing *pykF* [13]. These amino acids, derived from the POP node, hold a promising market future in biotechnology because of their broad-spectrum utilization.

L-Trp mainly serves as a food additive, pharmaceutical, and animal feed to improve sleep state and mood and promote animal growth [14], whereas L-Tyr and L-Phe are mainly utilized as materials for the generation of the anti-Parkinson’s disease drug L-dopa and the sweetener aspartame, respectively [15,16]. L-Val and L-Ile have been recently used as functional products and constituents of infusions [17]. In addition, L-Val is also used as a chemical synthon for antiviral drugs, antibiotics, and herbicides [18]. Essential amino acids, including L-Lys and L-Thr, play a pivotal role in the nutrition of humans and livestock. These amino acids are primarily employed as dietary supplements and feed additives to ensure adequate intake [19]. Additionally, L-Thr finds application as a starting material for the synthesis of high-value pharmaceutical intermediates [20]. Meanwhile, L-Lys serves as a precursor for ε-poly-L-lysine, a versatile compound utilized as a food preservative, drug delivery vehicle, and anti-obesity agent [21].

Their industrial-level production has become a significant challenge in supporting the essential applications of these amino acids [22]. Strain improvement is crucial for industrial production. Most utilized amino acid producers have been developed for decades via random mutagenesis and screening procedures [23]. However, this approach may retard cell growth and promote by-product accumulation. In addition, undefined mutations render it challenging to elucidate the high-yield mechanism, which obstructs subsequent strain improvement. The predominant methods for strain enhancement have recently been converted to incorporate rational metabolic engineering, which modifies pathway genes to increase the production of a target amino acid. Nevertheless, such small-scope alteration is insufficient to condense the metabolic flow toward producing desired amino acids. The development of systems biology has overcome this defect by utilizing omics information and biochemical analysis [24].

This review focuses on summarizing the current biosynthesis pathways of seven POP-AAs, exploring the metabolic engineering techniques utilized in *C. glutamicum* and *E. coli* to boost their production efficiency. Furthermore, we put forward a comprehensive metabolic engineering strategy that integrates systems biology, synthetic biology, and molecular modifications as an innovative approach to enhance the generation level, yield, and overall productivity of these amino acids further. The specific POP-AA production methods and associated engineering strategies are concisely listed in Table 1 and Table 2.

## 2. L-Tryptophan

Three sequential biosynthetic pathways are used for L-Trp formation [89]. Glycolysis and the pentose phosphate pathway (PPP) constitute the common metabolic pathways for L-Trp. Through glucose metabolism, two moles of PEP are produced by glycolysis. Utilizing glucose as the sole carbon source, a minimal medium supports the growth of *E. coli*, while 50% of the PEP produced from glycolysis is utilized as a phosphorous source for the phosphotransferase system, regulating the import of glucose. Moreover, 32% of the PEP is catalyzed to PYR by PYK and OAA by PEP carboxylase (PEPC). However, only 3% of the PEP is available for aromatic amino acid biosynthesis, leading to PEP limitation in L-Trp production [25]. Therefore, engineering the POP node is a fascinating target for improving L-Trp production. In addition, in the PPP, both oxidative and nonoxidative pathways can be used to synthesize erythrose 4-phosphate (E4P). Although oxidative PPP generation of NADPH can be more advantageous than its generation via nonoxidative PPP, it is accompanied by the release of 1 mol carbon dioxide for every molecule of G6P broken down [26]. PEP and E4P are then metabolized to generate 3-deoxy-D-arabino-heptulosonate-7-phosphate (DAHP).

**Figure 2:**
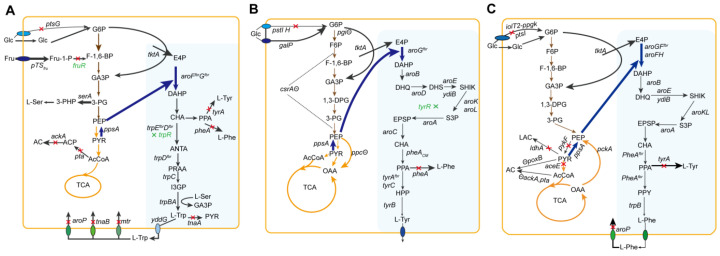
Engineering strategies for enhancing PEP-node-derived amino acid production. Metabolic flux of (**A**) L-Trp, (**B**) L-Tyr, (**C**) L-Phe. Letters in green indicate an inhibitor of the enzyme. The cross mark indicates the gene is knocked out. The double colon indicates the gene is replaced. The minus symbols indicate activation and repression of the gene expression, respectively. Abbreviations: 1,3-DPG, glycetate-1,3-diphosphate; 3-PG, glycerate-3-phosphate; 3-PHP, hydroxypyruvate-3-phosphate; ACP, acetyl phosphate; ANTA, anthranilate; CHA, chorismate; DAHP, 3-deoxy-D-arabinoheptulosonate-7-phosphate; DHQ, 3-dehydroquinate; DHS, 3-dehydroshikimate; E4P, erythrose-4-phosphate; EPSP, 5-enolpyruvoylshikimate; F-1,6-BP, fructose-1,6-bisphosphate; F6P, fructose-6-phosphate; GA3P, glyceraldehyde-3-phosphate; HPP, 4-hydroxyphenylpyruvate; I3GP, indole 3-glycerolphosphate; PPA, prephenate; PPY, phenylpyruvate; PRAA, phosphoribosyl anthranilate; S3P, shikimate-3-phosphate; SHIK, shikimate; *ackA*, acetate kinase; *aroA*, 3-phosphoshikimate-1-carboxyvinyltransferase; *aroB*, 3-dehydroquinate synthase; *aroC*, chorismate synthase; *aroD*, 3-dehydroquinate dehydratase; *aroE*, shikimate dehydrogenase isozyme; *aroF^fbr^G^fb^*^r^, feedback-resistant 3-deoxy-D-arobino-heptulosonate synthase; *aroH*, 3-deoxy-D-arobino-heptulosonate synthase; *aroK*, shikimate kinase I; *aroL*, shikimate kinase II; *aroP*, aromatic amino acid exporter; *fruR*, fructose repressor; *galp*, galactose permease; *iolT2*, major myoinositol transporter IolT; *ldhA*, lactate dehydrogenase; *mtr*, L-tryptophan importer; *pckA*, phosphoenolpyruvate carboxykinase; *pgi*, phosphoenoglucose isomerase; *pheA*, chorismate mutate-prehenate dehydratase; *poxB*, pyruvate oxidase; *ppc*, phosphoenolpyruvate carboxylase; *ppgk*, polyphosphate glucokinase; *ppsA*, phosphoenolpyruvate synthase; *pta*, phosphotransacetylase; *pTS_fru_*, frucose-specific phosphotransferase system; *ptsG*, fused glucose-specific phosphotransferase system enzyme IIBC; *ptsH*, phosphotransferase system phosphohistidinoprotein-hexosephosphotransferase (HPr); *ptsI*, phosphoenolpyruvate-protein phosphotransferase of phosphotransferase system; *pykF*, pyruvate kinase; *tktA*, transketolase; *tnaA*, tryptophanase; *tnaB*, L-tryptophan importer; *trpBA*, tryptophan synthase; *trpC*, indole glycerol phosphate synthase; *trpE^fbr^D^fbr^*, feedback-resistant anthranilate synthase; *trpR*, trp operon repressor; *tyrA*, chorismate mutate-prehenate dehydratase; *tyrB*, tyrosine aminotransferase; *tyrC*, cyclohexadienyl dehydrogenase; *yddG*, aromatic amino acid exporter; *ydiB*, shikimate dehydrogenase isozyme.

The second pathway constitutes the common aromatic route, in which DAHP is converted to chorismate (CHA). The pathway has no competition routes for shunting; rather, its rate-limiting steps are regulated by feedback inhibition. The third pathway is the L-Trp branch route, in which CHA forms L-Trp through multiple steps (Figure 2A). Because anthranilate synthase (ANTAS) has a higher affinity to CHA than CHA mutase/prephenate dehydrogenase or CHA/prephenate dehydratase [90]. CHA is preferred for L-Trp synthesis. However, the *trp* operon is regulated by transcriptional repression and attenuation, and L-serine is needed as well. The accumulated L-Trp is secreted extracellularly through the exporter carrier YddG, which can also export both L-Tyr and L-Phe [91]. With Mtr and TnaB having high and low affinity for L-Trp, they, as well as AroP, control the import of L-Trp [27]. However, only one transporter, AroP, controls the import of L-Trp in *C. glutamicum* [92].

So far, the primary metabolic engineering strategies aimed at enhancing L-Trp production have encompassed the following: (1) modification of core biosynthetic pathways, (2) optimization of carbon flux, (3) engineering of transport systems, (4) elimination of undesirable by-products, and (5) combined high-throughput screening (HTS) and multidimensional regulation. Herein, we highlight the main research advancements achieved in recent years in the production of L-Trp through the utilization of engineered *E. coli* and *C. glutamicum*.

### 2.1. Engineering the Common Pathway and L-Trp Branch for Enhanced L-Trp Production

Previous L-Trp development research has most often focused on the inactivation of the trp operon repressor trpR and tryptophanase tnaA, followed by expressing feedback-resistant DAHP synthase, ANTAS, trp operon, and 3-phosphoglycerate dehydrogenase (PGD). Schoppel et al. obtained the *E. coli* NT1439 by the combination of the genomic overexpression of *trpBA* and *trpC_mt_*; the total L-Trp amount was increased to 401.0 g in the fed-batch process on a 15 L scale [93]. The L-Trp biosynthesis pathway was further enhanced by overexpressing the L-Trp operator sequences (*trpEDCBA*) and 3-deoxy-D-arabinoheptulosonate-7-phosphate synthase (*aroG*^fbr^). The engineered strain *E. coli* KW023 produced 39.7 g/L of L-Trp with a conversion rate of 16.7% and a productivity of 1.6 g/L/h in a 5 L fed-batch fermentation [94]. JB102, a *tnaA* and PGD gene *serA* mutant, was engineered to co-express *trpE^fbr^DCBA*, the DAHP synthase gene *aroG^fbr^*, and *serA* on pBE7, improving L-Trp production to 42.3 g/L with a yield of 0.176 g/g glucose by controlling the feed rate [95]. Chen and his team unveiled a groundbreaking regulatory framework governing the L-Trp biosynthetic pathway [96]. Specifically, they observed that the indole-glycerol phosphate synthase, a component of the multifunctional enzyme TrpC in *E. coli*, undergoes a non-competitive feed-forward inhibition triggered by anthranilate. By introducing the anthranilate-activated TrpC variant from *A. niger* into a previously modified L-Trp-producing strain of *E. coli*, namely S028, they were able to enhance the L-Trp production significantly, achieving a titer of 29 g/L in simplified fed-batch fermentations. Although the L-Trp-overproducing strains have been modified by integrating genetic engineering strategy with random mutagenesis, the genome of these strains still retains some undesirable mutations. Therefore, further strain construction would be limited.

In a recent study, *E. coli* producers developed with a defined genetic background in a wild-type strain via genetic engineering were utilized to generate L-Trp. Strain FB-04 originated from *E. coli* W3110, which contains the overexpressed genes for DAHP synthase (*aroF^fbr^*) and anthranilate synthase (*trpE^fbr^D*) on pSV, along with deletion of *trpR*-*tnaA* and L-Phe and L-Tyr biosynthesis pathway rate-limiting enzyme genes (*pheA* and *tyrA*). The strain produced 13.3 g/L L-Trp at a yield of 0.1 g/g glucose after 40 h by fed-batch cultivation [97]. TRTH0709, which also originated from *E. coli* W3110, contains overexpressed genes in the tryptophan operon (*trpE^fbr^DCBA*) and for DAHP synthase (*aroG^fbr^*), along with the serine-biosynthetic gene (s*erA*) on pBR322, with deletion of *trpR* and *tnaA*, improving L-Trp production to 35.9 g/L [28].

Additionally, an L-Trp producer, *C. glutamicum* KY10894, was engineered to co-express the deregulated DAHP synthase gene and trp operon. The developed strain improved L-Trp yield by 54% at a final level of 43 g/L, with indole accumulated as a by-product [98]. Further expression of *serA* from ATCC 31833 eliminated indole accumulation and enhanced L-Trp yield by 61% at a titer of 50 g/L, with a productivity of 0.63 g/L per hour [25]. Drawing upon the previously developed L-Trp-producing strain *E. coli* T8, Liu et al. achieved a significant improvement in the titer and yield of L-Trp via combining three metabolic engineering techniques. Firstly, they targeted the shikimate pathway and the L-Trp biosynthetic branch, analyzing and deciphering the rate-limiting steps to overexpress the crucial genes *aroE* and *aroD*. Then, they effectively improved the intracellular availability of the precursors L-glutamine and L-serine and finally modulated the transport system and competitive pathway of L-Trp through the elimination of the gene *TnaB*. Following 42 h of fed-batch fermentation, the modified strain *E. coli* T13 produced 53.65 g/L of L-Trp with a yield of 0.238 g/g glucose [29].

### 2.2. Engineering the POP Node and PPP to Improve Carbon Flux for PEP and E4P Formation

PEP and E4P comprise two core chemicals in L-Trp production, acting as crucial precursors in the shikimic acid pathway. Engineering the POP node and PPP key enzyme, including PEP synthase, glucose phosphotransferase system (PTS), PYK, and transketolase (TktA) genes, can reduce pyruvate production to increase PEP and E4P formation. Amplifying the PEP synthase gene *ppsA* alone in *E. coli* TRTH0709 does not significantly increase L-Trp yield. In comparison, amplification of the TktA gene *tktA*, alone or in conjunction with *ppsA*, produced L-Trp at a yield of 37.9 and 40.2 g/L, respectively, representing a 5.6% and 11.9% increase compared to that of the original strain [93]. Deletion of PYK I gene *pykF* in an L-Trp-producing strain, *E. coli* FB-04(*pta1*), showed a slightly higher L-Trp yield and a higher conversion rate. However, the phosphocarrier protein (Hpr) gene *ptsH* was deleted in the engineered strain, and the levels of biomass, L-trp yield, and conversion rate of this strain were deficient during fed-batch fermentation. To restore the impaired growth and glucose utilization capabilities resulting from the *ptsH* deletion, four specific mutations (N12S, N12A, S46A, and S46N) of Hpr protein were strategically introduced into the chromosome of *E. coli* FB-04(*pta1*) Δ*pykF*/*ptsH*. Notably, the N12S mutation variant demonstrated exceptional performance, exhibiting a glucose conversion efficiency of 0.178 g/g, representing a notable enhancement of 38.0% compared to the original strain [99]. *E. coli* MA01 (Δ*tnaA*, Δ*serA*, [pBR322-*aroG*^fbr^-*trp*^fbr^*EDCBA*]) was engineered to disrupt *ptsG* to increase PEP production via the Red homologous recombination system. *E. coli* MA209 produced 33.4 g/L L-Trp at a glucose converse rate of 15.5%. Another strain, *E. coli* MA103, in which the PTS gene *ptsHIcrr* was deleted, and the glucose facilitator-glucokinase genes *glf* and *glk* were overexpressed from the plasmid, produced 38.5 g/L L-Trp at glucose converse rate of 16.7%. However, the by-product acetate was also increased in this strain because the expression levels of *glf* and *glk* were high [100]. Two genes were first disrupted, including *pykA* and the PEP carboxylase gene *ppc*. An engineered *E. coli* expressed the PEP carboxylase gene *pck*, citric acid transport gene *citT*, aconitase gene *acnBA*, isocitrate dehydrogenase gene *icd*, and pyruvate carboxylase gene *pyc* via chromosomal integration or promoter replacement. L-Lrp production increased to 49 g/L at a yield of 0.186 g/g glucose in a 5 L bioreactor fed-batch fermentation [30]. In addition, knocking out the gene encoding the fructose repressor FruR (*fruR*), a regulatory factor of the glycolysis synthesis route of *E. coli*, in the engineered strain *E. coli* FB-04 (*Δtrp*, *ΔtnaA*, *ΔpheA*, *ΔtyrA*[pACYC177-*aroF*^fbr^-*trpE*^fbr^*D*]) increased L-Trp titer and yield by 62.5 and 52.4%, respectively, compared to those of the parental strain [89]. Metabolic flux analysis revealed that the *fruR* deletion markedly increases carbon flux via glycolysis and the PPP, improving levels of the critical starting materials PEP and E4P for L-Trp generation.

A classically derived L-Trp producer, *C. glutamicum* KY9218 carrying pKW9901, was developed to synthesize higher levels of L-Trp. According to the carbon balance analysis, the TktA gene *tktA* from *C. glutamicum* was co-amplified in the recombinant bacteria by utilizing the low-copy plasmid pIK9960. The L-Trp production of the final strain was 58 g/L, representing a 15% increment over the original strain, as the highest production level ever reported [26]. To address the imbalance in flux distribution between central carbon metabolism and L-Trp biosynthesis in *E. coli*, Xiong et al. enhanced PEP and E4P pools by introducing a phosphoketolase from *Bifidobacterium adolescentis* and a modified sugar transport system, which substituted the original with a glucose facilitator from *Zymomonas mobilis* and a natural glucokinase. PEP–pyruvate–oxaloacetate nodes were also rewired to redirect the central carbon flux towards the L-Trp biosynthesis pathway. The resulting strain produced 41.7 g/L L-Trp in 40 h with a yield of 0.227 g/g glucose, demonstrating the importance of sufficient PEP and E4P for L-Trp biosynthesis [31].

### 2.3. Combined Transport Engineering and By-Product Elimination

Although feedback-resistant mutated enzymes have been obtained, it remains difficult to completely deregulate allosteric inhibition of the critical enzymes in the L-Trp synthesis route. Accordingly, transport engineering represents an essential strategy for strain improvement through bypassing the remaining feedback regulations. Four transporters, Mtr, TnaB, AroP, and YddG, participate in importing and exporting L-Trp in *E. coli*. Following individual deletion of the permease genes *mtr*, *tnaB*, and *aroP* in an L-Trp producer *E. coli* FB-03, *mtr* was found to constitute the most crucial component of the L-Trp transport machinery for L-Trp import and synthesis. Specifically, the *mtr* deletion mutant generated 14.7 g/L L-Trp at a yield of 0.12 g/g glucose [101]. By manipulating the *aroP* gene deletion and YddG gene overexpression in the L-Trp-producing strain *E. coli* TRTH, the glucose conversion rates of the engineered strains reached 5.2% and 6.2%, respectively, surpassing the parental strain’s rate of 4.4% in flask-shaking culture conditions. However, unexpectedly, the glucose conversion rate of the *E. coli* TRTH Δ*aroP*-Y strain stood at 4.6%, failing to demonstrate an anticipated increase. This was attributed to the constrained availability of endogenous aromatic amino acids, which are essential for protein biosynthesis. When fed-batch fermentation was used in *E. coli* TRTH-Y, the L-Trp production level increased by 12.6% at a final titer of 36.3 g/L in a 30 L fermentor [102]. Furthermore, Gu et al. disrupted three transporter genes simultaneously in the L-Trp producer *E. coli* GPT-1002 (*ΔtrpR*, *ΔtnaA*, *ΔptsG*, *Δmtr*, P*trp*::5CP*tacs*, [pCL1920-*aroG*^fbr^-*trpE*^fbr^-*tktA*]) [32]. The triple-gene disruption mutant *E. coli* 1017 achieved L-Trp formation at 16.3 g/L, 50% more than that generated using the parent strain in 5 L fermentor cultivation.

However, *E. coli* strains commonly produce a large amount of acetate by-product during fermentation. The Pta-AckA route comprises the main acetate generation route. Phosphate acetyltransferase (Pta) and acetate kinase (AckA), encoded by genes *pta* and *ackA*, respectively, produce acetate from acetyl-CoA via two successive reactions. Inactivation of *pta* significantly improved L-Trp synthesis compared to that synthesized by inactivation of *ackA* in *E. coli* FB-04. Nevertheless, FB-04(*Δpta*) demonstrated inhibited cell growth. When *pta* was substituted with its variant *pta*1(P69L) from *E. coli* CCTCC M2016009, a 15% improvement in the L-Trp generation was achieved, up to 44.0 g/L [103]. Deletion of the *pta*–*ackA* genes in a *gltB* (encoding glutamate synthase) mutant of *E. coli* TRTHB inhibited acetate formation and increased L-Trp yield by 17.83%, at a titer of 47.18 g/L. When a novel cell recycling strategy, 1:1 concentrated cell solution/clear solution with cell recycling within 24–30 h, was used to separate the product and harmful intermediates continuously, the engineered strain improved L-Trp production to a titer of 55.12 g/L and glucose converse rate by 19.75% [104]. In addition, deletion of *ptsG* in the L-Trp-producing strain *E. coli ΔtrpR Δtna*, *aroG*^fbr^*trpE*^fbr^*DCBA* showed better results than mutants with deletions of either *pta* or *pta*-*ptsG*. Based on suitable dissolved oxygen levels, glucose feedback feeding, and sequential pH adjustment strategies, the mutant strain increased L-Trp yield by 35.81% with a titer of 17.14 g/L, with acetate yield reduced by 71.08% [105]. The combination of by-product acetate elimination and the modification of the TCA cycle improved L-Trp production. The gene *acs*, involved in the acetate-acetyl CoA cycle and encoding acetyl CoA synthase, was integrated into the *araB* site by the *ara* promoter in *E. coli* TRTH; the by-product acetate reduced by 38.1%, but the L-Trp produced decreased by 15.2% owing to insufficient energy. Further integrating genes P*ara*-*aceB*-*mdh*-*pck* into the *yghx* site created a final strain that improved L-Trp production by 11.4% at a titer of 54.6 g/L [33].

The integration of transport engineering with acetate by-product elimination further enhanced L-Trp synthesis. The *pta* and *mtr* genes were disrupted in the chromosome of *E. coli* TRTH0709, and the aromatic amino acid transport gene *yddG* was overexpressed from a plasmid. The resultant strain synthesized L-Trp at 48.68 g/L after 35 h with a yield of 0.22 g/g glucose, which corresponds to 43% of the theoretical maximum [34].

### 2.4. Combined High-Throughput Screening (HTS) and Multidimensional Regulation

Given the complexity of the physiological and metabolic network systems within microorganisms, the challenges of establishing a more effective cell factory using traditional approaches have significantly escalated. As an alternative, the development of an HTS platform emerges as a viable solution for the efficient acquisition of targeted phenotypic traits [106]. Concurrently, biosensors have emerged as invaluable tools for HTS, metabolite quantification, and dynamic regulation [107]. Specifically, the riboswitch-driven biosensor has been engineered to regulate metabolic fluxes [108] and serve for HTS [35], thereby enhancing amino acid biosynthesis.

In a recent study, Tang et al. successfully developed a riboswitch-based HTS platform, which was instrumental in the construction of a robust chassis cell for the production of L-Trp. The final strain *E. coli* Trp30 based on this platform yielded remarkably favorable outcomes, wherein in a 5 L bioreactor, it achieved a titer of 42.5 g/L for L-Trp and a yield of 0.178 g/g glucose after 48 h of cultivation without the need for additional supplements. The significant accomplishment was primarily due to the integration of HTS with multidimensional metabolic engineering techniques, encompassing the modification of promoter and N-terminal coding sequences (NCSs); precise adjustments to degradation, transport, and by-product synthesis routes; transcription factor engineering; dynamic regulation of branching pathways; and augmentation of cofactor availability. As a result, the L-Trp-producing strain Trp30 attained an unprecedented production level, surpassing all previously reported strains that lacked plasmids and additional additives [35]. Collectively, the strategies outlined above represent a promising methodology that combines HTS with multidimensional regulation for the development of efficient cell factories for L-Trp production.

## 3. L-Tyrosine

L-Tyr comprises one of three aromatic amino acids biosynthesized through the common shikimate pathway, originating from PEP and E4P, ultimately leading to the formation of CHA. CHA is metabolized to L-Trp and L-Phe by anthranilate synthase and CHA mutase (Cm)-prephenate dehydratase (Pdh), respectively. L-Tyr is formed via three reactions from CHA (Figure 2B). First, Cm catalyzes CHA into prephenate. Prephenate is then catalyzed by Pdh to form 4-hydroxyphenylpyrvate (HPP), which is further catalyzed into L-Tyr through glutamic acid transamination with aminotransferase. In *E. coli*, L-Tyr restricts the activity of Cm, and TyrR combines with L-Tyr to inhibit the transcription of the Cm-Pdh-coding gene *tyrA*. In *C. glutamicum*, the activity of CM is feedback-inhibited by L-Phe and L-Tyr. Synthesized L-Tyr in the cell is secreted through the aromatic amino acid exporter YddG [91]. Two systems control the uptake of L-Tyr: the common aromatic import system AroP and the specific import system TyrP.

The work of Lütke-Eversloh described the production of L-Tyr using deregulated strains of *Escherichia coli* in 2007 [36]. Several publications have recently employed L-Tyr overproducers to synthesize L-Tyr-derived compounds [109]. Meanwhile, of the three aromatic amino acids originating from the shikimate pathway, the yield of L-Tyr ranges from 0.10 to 0.44 g/g glucose. Although Patnaik et al. described L-Tyr levels of more than 50 g/L in a 200 L bioreactor by strengthening the cultivation and separation strategies in *E. coli* [37], only 0.09 g/g glucose of L-Tyr was yielded in a 3-(N-morpholino) propane sulfonic acid (MOPS) minimal medium [38], which is less than 20% of the theoretical yield.

The prevailing strategies for enhancing L-Tyr production via metabolic engineering encompass the following: (1) modification of core biosynthetic pathways, (2) elimination of competing pathways, (3) cofactor engineering, (4) implementation of global and modular metabolic engineering approaches, and (5) development of glucose–xylose–phenolic (GXP) systems for L-Tyr production from lignocellulosic biomass. As metabolic engineering techniques continue to be introduced, the yield of L-Tyr has progressively increased. Herein, we elaborate on the main research advancements achieved in the metabolic engineering of *E. coli* and *C. glutamicum* for the production of L-Tyr.

### 3.1. L-Tyr Biosynthetic Pathway Modification, Competition Pathway Elimination, and Cofactor Availability Improvement

As the genomes of *E. coli* and *C. glutamicum* have been deciphered, the biosynthetic route of L-Tyr has become the primary target for enhancing L-Tyr synthesis. The key enzymes in the route are sensitive to feedback inhibition and transcriptional attenuation by end products and TyrR, respectively. Overexpressing the critical genes involved in the biosynthetic route, particularly the alleviated ones, generally represents the most efficient method. For example, overexpression of cyclohexadienyl dehydrogenase (TyrC) from *Zymomonas mobilis* and the Cm domain of natural CHA mutase prephenate dehydratase (PheA_CM_) from *E. coli* in the PTS**^−^** glucose**^+^** strain was utilized to deregulate the L-Tyr inhibition of Cm-Pdh. The reconstructed *E. coli* PB12CP generated 3 g/L L-Tyr at a yield of 0.066 g/g glucose [110]. In another study, feedback inhibition was relieved by the co-expression of the L-Phe 4-hydroxylase from *Xanthomonas campestris* and the tetrahydromonapterin recycling enzymes in *E. coli* BW25113 to divert carbon flux from L-Phe to L-Tyr. The recombinant strain *E. coli* JH7 produced L-Tyr at a titer of 262 mg/L. Further module expression of the shikimate pathway rate-limiting enzymes AroL and AroG^fbr^, precursor biosynthetic enzymes PpsA and TktA, and tetrahydromonapterin biosynthetic enzymes FolX and MtrA increased the L-Tyr production to 401 mg/L in shake flask culture [39]. Similarly, a recombinant *E. coli* was developed to express *aroG^fbr^* and *tyrA^fbr^*. The *tyrR* gene, encoding TyrR protein, was also deleted in this strain. L-Tyr production by the strain reached 3.8 g/L in 22 h with a yield of 0.037 g/g glucose [36]. Further expression of *ydiB*, encoding shikimate dehydrogenase, and *aroK*, encoding shikimate kinase I, increased the final L-Tyr level by 45% in *E. coli* T1 [111]. Strain *E. coli* W3110 (*ΔtyrR*, *ΔtyrA*, *ΔpheA*), expressing feedback inhibition-resistant genes *aroG* and *tyrA* and shikimate kinase I-encoding gene *aroL*, achieved an L-Tyr level of 6.3 g/L at a yield of 0.16 g/g glucose [40]. Also, *aroG*^fbr^-*aroL* and *tyrC* were co-expressed inductively under the *Ptac* and *ptrc* promoter in BL21(DE3), respectively. Further deletion of the L-Tyr-specific transporter (tyrP) improved L-Tyr production to 43.14 g/L and a yield of 0.107 g/g glucose using the exponential-to-DO-stat feeding strategy [41].

An L-Trp *C. glutamicum* producer was also developed to synthesize L-Tyr. Drawing from the aromatic amino acid metabolic regulation model, Ping et al. recently utilized a synergistic engineering strategy in *E. coli* to bolster L-Tyr production. Specifically, they overexpressed two key biosynthesis enzymes: DAHP synthase, a common pathway enzyme, and the branch-point enzyme CM, which was deregulated to circumvent product inhibition, resulting from genetic mutations in *C. glutamicum*. This approach was implemented in the L-Trp-producing strain KY10865, leading to an L-Tyr yield of 26 g/L [112]. In a parallel effort, an L-Tyr-overproducing *E. coli* strain, DPD4193, was derived from the L-Phe overproducer NST37. This strain achieved remarkable performance by deleting the *pheA* gene encoding CHA mutase-prephenate dehydratase and its regulatory gene *pheL*, while overexpressing *tyrA* via promoter exchange. This strain yielded L-Tyr at a high titer of 55 g/L, with a yield of 0.3 g/g glucose in a 200 L batch culture [37,38].

Gold et al. introduced a novel strategy to enhance L-Tyr production in *Saccharomyces cerevisiae*. Their approach targeted two regulatory sites: DAHP synthase and cofactor limitation at prephenate dehydrogenase. By knocking out phenylpyruvate decarboxylase Aro10 involved in the Ehrlich pathway and overexpressing feedback-resistant enzymes AroGK299L and TyrC from Zymomonas mobilis, they were able to redirect metabolic flux towards L-Tyr production. Further, by deleting the *ZWF1* gene encoding glucose-6-phosphate dehydrogenase and expressing NADP^+^-dependent prephenate dehydrogenase Tyr1, they compelled the cell to couple its growth with L-Tyr synthesis. This resulted in an engineered strain, Zwf1^-^, capable of producing 520 μmol/g cells of L-Tyr in the presence of L-methionine [112].

In a recent investigation, Ping and colleagues applied a synergistic engineering methodology in *E. coli* to optimize the productivity of L-Tyr. This strategy primarily centered on the modulation of expression patterns for key enzymes within the shikimic acid pathway, which was complemented by transport engineering. Furthermore, modifications to the acetic acid biosynthesis pathway, the introduction of the phosphoketolase pathway, and cofactor engineering were also integrated. Following these comprehensive genetic modifications, adaptive laboratory evolution was employed to select the optimal acid-tolerant strain, named *E. coli* HGD (M9). This strain demonstrated remarkable performance, achieving a production titer of 92.5 g/L L-Tyr in a 5 L fermenter with a yield of 0.266 g/g glucose in 62 h, the highest titer reported so far [43].

### 3.2. Combined Regulation of the PPP and POP Node

Once bottlenecks in the L-Tyr biosynthetic pathway are removed, increasing precursor PEP and E4P supplementation constitutes an effective strategy for L-Tyr production. The endogenous *tktA* gene was overexpressed in the L-Tyr-producing strain *C. glutamicum* KY10621 to increase PPP flux for L-Tyr production. The recombinant strain accumulated 6.3 g/L L-Tyr within 72 h in a shake flask culture [113]. The PTS gene *ptsIH* was deleted to cut off carbon flux from PEP to PYR and increase the expression of galactose permease gene *galP* via promoter exchange to increase glucose internalization. The recombinant *E. coli* strain VH33*ΔtyrR-*TIR produced L-Tyr with a yield of 0.36 g/g glucose, representing 66% of the theoretical value [114]. In addition, the 5′-untranslated region (5′-UTR) was replaced by the nascent genetic element of PEP synthase to rebalance the metabolic flow at the POP node using UTR Designer. The constructed *E. coli* synthesized 3 g/L L-Tyr, with productivity of 0.034 g/L per hour and a yield of 0.102 g/g glucose [113].

The combination of PPP flux and POP node enhancement improved L-Tyr production. TktA was also expressed in combination with PEP synthase in the L-Tyr producer *E. coli* T1 to promote the availability of the two starting materials, PEP and E4P. The reconstructed strain *E. coli* T2 produced 9.7 g/L L-Tyr at a yield of 0.102 g/g glucose in 3 L fed-batch fermentation [36]. Further overexpression of the acetyl-CoA synthetase gene *acs* and PEP carboxykinase gene *pck* via the strong constitutive promoter (P_J23100_) and synthetic 5′ UTR induces L-Tyr production from acetate. Additionally, the isocitrate lyase gene *aceA* was precisely regulated by different-strength promoters to balance metabolic flux between glyoxylate and TCA cycles for enhancing PEP availability and the generation of ATP and NADH. The final strain, *E. coli* SCKAPG4, produced L-Tyr at 0.70 g/L, with a productivity of 0.023 g/L per hour [115].

### 3.3. Global Metabolic Engineering for Enhanced L-Tyr Formation

Previous L-Tyr development research has primarily concentrated on only a few candidate genes of the L-Tyr route per study, requiring the selection of numerous strains to avoid defects. Although this strategy can promote carbon flux toward these routes, it is tedious and time-consuming. Recently, global metabolic engineering has been developed for L-Tyr improvement through the precise regulation of L-Tyr synthetic pathway gene expression via modular engineering, global transcription machinery engineering (gTME), and synthetic small regulatory RNAs (synthetic sRNAs). Juminaga et al. divided L-Tyr biosynthetic genes into two modules by modular engineering, one harboring six genes for the accumulation of shikimate from PEP and E4P and another carrying five genes for the final generation of L-Tyr from shikimate [116]. The obstacles in the two modules were solved by alteration of gene codon usage, promoter strength, plasmid copy number, and the placement of genes in operons to generate two individual modules. Various shikimate and L-Tyr modules were assembled into the original strain *E. coli* MG1655 with the aim of decreasing shikimate production and coordinating gene expression to increase L-Tyr formation. The module assemblages P_LacUV5_ and P_LtetO-1_ for regulating *aroE*-*aroD*-*aroB* and *aroG^fbr^*-*ppsA*-*tktA* and P_LacUV5_ and P_Trc_ for regulating *tyrB*-*tryA^fbr^*-*aroC* and *aroA*-*aroL* performed best, increasing L-Tyr production to 80% of the theoretical yield with a titer of 2.6 g/L.

In turn, *E. coli* strain rpoA14^R^ was derived from P2 through error-prone polymerase chain reaction (PCR) using gTME. The provision of one mutation, at the site encoding RNA polymerase subunit rpoA (V257F, L281P), on a plasmid altered the affinity of the RNA polymerase to its target genes. Another mutation site, encoding imidazole glycerol phosphate synthase subunit mutant (L82R) in the chromosome, altered the affinity of the imidazole glycerol phosphate synthase for glutamine. The strain produced 13.8 g/L L-Tyr in 36 h at a high productivity of 2.1 g/L per hour in a 2 L fermentor [117].

Finally, the influence of synthetic sRNAs on L-Tyr synthesis has been evaluated through the combined overexpression of genes for synthetic sRNA-based inhibition, including TyrR (*tyrR*), carbon-storage regulator (*csrA*), phosphoenoglucose isomerase (*pgi*), and PEP carboxylase (*ppc*). Among these sRNA integrations, anti-*tyrR* and anti-*csrA* in *E. coli* S17-1 synthesized the highest L-Tyr level at 2 g/L. Moreover, when the high-density culture was utilized in this strain, production improved to 21.9 g/L [44].

### 3.4. Development of Glucose–Xylose–Phenolic (GXP) System for L-Tyr Production from Lignocellulosic Biomass

Lignocellulosic biomass represents the world’s most abundant renewable resource, offering significant potential for the production of sustainable fuels and chemicals as viable alternatives to petrochemicals, which have detrimental effects on the global climate [118]. Zhao et al. pioneered the development of a GXP system that efficiently utilizes lignocellulose for the microbial biosynthesis of L-Tyr. This innovation not only highlights the substantial cost-reduction potential in the production process but also lays a solid foundation for further optimizations [119].

Given that the lignocellulose hydrolysate primarily consists of glucose, xylose, and lignin-derived phenolics, a microbial consortium capable of utilizing these three compounds as carbon sources was constructed. Initially, an *E. coli–E. coli* consortium harboring CRISPR/dCas9 was assembled, and it efficiently utilized glucose and xylose to sustain cell growth while concomitantly producing pyruvate. Subsequently, the introduction of tyrosine phenol lyase and other crucial biocatalysts culminated in the completion of the system, enabling it to effectively convert pyruvate and lignin-derived phenolics into L-Tyr. Notably, using the hydrolysate derived from sorghum pith as a feedstock, a total yield of 0.163 g of L-Tyr per gram of raw material was achieved [119]. This successful demonstration underscores the viability and advantages of employing the GXP system for harnessing lignocellulose biomass for microbial L-Tyr production. This achievement holds immense potential for promoting the sustainable utilization of renewable resources and advancing the field of bioproduction.

## 4. L-Phenylalanine

L-Phe biosynthesis is divided into three parts [120]. The first is central carbon metabolism, in which glucose is catabolized to PEP and E4P through glycolysis and PPP, respectively. The second part involves the shikimate pathway, wherein central carbon intermediates PEP and E4P are condensed to form DAHP. In *E. coli*, three DAHP synthases encoded by *aroF*, *aroG*, and *aroH* catalyze a specific reaction. These enzymes, known as isozymes, are individually influenced by transcriptional and allosteric regulation triggered by L-Tyr, L-Phe, and L-Trp. DAHP is then catalyzed to CHA, the branch node of aromatic amino acid generation. The third part is the CHA pathway. L-Phe is generated from CHA by three successive steps metabolized by Cm-Pdh and aromatic amino acid transaminase (Figure 2C). Cm-Pdh, encoded by the *pheA* gene, is susceptible to feedback inhibition through allosteric binding of L-Phe. Additionally, its expression is modulated by operator-mediated repression and attenuation mechanisms.

The prevailing strategies for enhancing L-Phe production via metabolic engineering encompass the following: (1) optimization of carbon flux, (2) elimination of competitive pathways as well as by-products, (3) engineering to strengthen precursor pathways, (4) modular metabolic engineering approaches, (5) regulator redesigning, and (6) adaptive laboratory evolution. While *E. coli* and *C. glutamicum* represent candidate L-Phe-producing strains, their production level and yield are low. Through random mutagenesis and genetic engineering, the yield of the L-Phe-producing engineered strain has progressively increased [45]. However, the molecular mechanism by which this strain synthesizes a high titer of L-Phe has been partially elucidated, as this is more complex to understand than strain development achieved through rational metabolic engineering. Herein, we discuss key metabolic engineering advances in *E. coli* and *C. glutamicum* for enhanced L-Phe production.

### 4.1. Engineering to Improve Carbon Flux for L-Phe Production

Metabolic engineering to improve L-Phe productivity through the overexpression of critical enzymes involved in shikimate and chorismate biosynthesis has achieved some success. Overexpression of *aroF^fbr^* and *pheA^fbr^* in *E. coli* produced L-Phe at 50 g/L after 36 h with a yield of 0.25 mol/mol [121]. Nevertheless, Gerigk et al. described that with the aid of L-Tyr and glucose control, native *aroF* could be utilized for L-Phe synthesis with higher L-Phe levels (34 g/L) than those achieved by the *aroF^fbr^* strain (28 g/L) by supplying greater DAHP synthase activity [122]. Subsequently, *E. coli* strain BR-42 originating from WSH-Z06 was screened through random mutagenesis for tolerance against bacteriophage BP-1. When expressing the deregulated Cm-Pdh gene *pheA^fbr^* and wild-type DAHP synthase gene *aroF^wt^*, an engineered strain BR-42/pAP-B03 produced L-Phe at 57.63 g/L at a productivity of 1.15 g/L per hour via a two-stage feeding strategy [45]. Quantitative proteomics integrated with multi-enzyme reaction system in vitro analysis identified that AroL and AroA overexpression would enhance L-Phe synthesis in *E. coli* HD-1. When the *AroL* gene was expressed in this strain, it produced L-Phe at 62.47 g/L and a yield of 0.236 g/g glucose, with the productivity of 1.3 g/L per hour in a 5 L fermentor by fed-batch fermentation [123]. In addition, the wild-type strain *E. coli* W3110 was constructed to express *pheA^fbr^* and *aroG^fbr^* to promote the metabolic flux to synthesize L-Phe, along with *ydiB^wt^* and *aroK^wt^* to improve the yield and shorten the lag phase. The strain generated 23.8 g/L L-Phe at a yield of 0.15 g/g glucose [124]. Furthermore, the shikimate pathway genes *aroG*^fbr^, *pheA*^fbr^, *aroALC*, and *aroEDB* were integrated into the *tyrR*, *ldhA*, *pykF*, and *ascF* loci of *E. coli* MG1655, utilizing a chromosome-based T7-dependent constitutive overexpression system. The constructed strain, M-PAR-200, produced 1.4 g/L L-Phe, with a productivity of 141 mg/g-DCW per hour and a yield of 0.14 g/g glucose [125]. However, traditional pathway enhancement often creates metabolic imbalances, a novel dynamic regulation strategy applied to increase L-Phe production. For example, dynamic regulation of Arok with the modified L-Phe-induced promoter P*_tyrP_* in the L-Phe-producing strain *E. coli* xllp1 in a time-dependent manner avoids metabolic imbalance. After 48 h of fed-batch fermentation, the mutant strain achieved an L-Phe titer of 61.3 g/L, yielding 0.22 g/g glucose. This represents a 1.36-fold increase in titer and a 1.22-fold increase in yield compared to previous results [46].

Similarly, marked improvements in L-Phe levels have been obtained by modifying the shikimate and chorismate pathway enzymes in *C. glutamicum*. An L-Phe producer was developed to express L-Phe-resistant *pheA^fbr^* from *E. coli*. The constructed strain KY10694/pEA22 increased L-Phe production by 35% at 23 g/L [120]. In comparison, co-amplification of the native gene *aroH* from *E. coli* and the mutated *pheA^fbr^* increased the level of L-Phe to 4.64 g/L [120]. Knocking tandem genes *aroG*-*pheA* into the *tyrA* site of *C. glutamicum* FP to delete the *tyrA* gene improved L-Phe yield by 71% with a level of 3.76 g/L [126]. Ikeda and Katsumata also developed an L-Trp producer *C. glutamicum* KY10865 strain for L-Phe generation [42]. DAHP was generated through PEP and E4P condensation by DAHP synthase and subsequently metabolized to CHA, branched to L-Trp, L-Tyr, and L-Phe. Overexpression of DAHP synthase and Cm, especially Pdh, redirected the carbon flow toward L-Phe. The engineered strain, KY10865/pKF1, produced L-Phe at 28 g/L by fed-batch cultivation [42].

### 4.2. Engineering to Weaken Competing Branches and By-Product Formation

Inhibition of the branching routes has been used to obtain more available carbon flow and decrease the generation of by-products. The integration of the ssrA-variant-tagged *tyrA* gene resulted in the engineered strain *E. coli* DV269 (TyrA-LAA) that produced less L-Tyr, although at amounts sufficient for cell growth. L-Phe synthesis by the strain achieved 7.2 g/L after 24 h with a yield of 0.14 g/g glucose [47].

The production of amino acids like L-Phe by microbes is often accompanied by the formation of by-products, including acetic acid. The stress-responsive gene *yggG* was integrated into the L-Phe-producing strain *E. coli* AJ12741 to inhibit acetic acid formation. The strain produced 6.4 g/L L-Phe at a yield of 0.16 g/g glucose. Further analysis showed that strain AJ12741/pHYGG also exhibited lower *pta*, *ackA*, and *poxB* expression levels [48].

### 4.3. Engineering to Strengthen Precursor Pathways

The influence of the POP node and PPP on L-Phe production has been evaluated via individual or combined overexpression of genes, including *ppsA*, *pckA*, and *tktA*, and deletion of the *PTS* gene. *PpsA* and the PEPC gene *pckA* were co-expressed on the same episomal vector as the *aroG*, *pheA*, and *tyrB* to enhance PEP supplementation. The constructed strain *B. flavum* 311 (pJN5) increased L-Phe yield by 2.4-fold with a 5.39 g/L [49]. Subsequently, the *csrA* gene was knocked into the chromosome of *E. coli* NST37, and the *tktA* gene was overexpressed from a plasmid to enhance E4P formation. The final strain yielded L-Phe with a 2.39 g/L [127]. In addition, TktA was expressed in combination with PTS deletion in *E. coli* PB12-ev2 (pJLB*aroG^fbr^* and pTrc*pheA^ev2^*). This strain showed improved PEP and E4P generation and produced L-Phe at a productivity of 40 mg/g-dcw per h, with a yield of 0.33 g/g glucose, corresponding to 60% of the maximum theoretical yield (0.55 g/g) [50].

### 4.4. Modular Engineering and Regulator Redesign for Enhanced L-Phe Production

The construction of recombinant strains commonly utilizes some engineering methods to condense the metabolism flux for L-Phe formation. Modular engineering constitutes a promising strategy. Toward this end, the critical genes in L-Phe biosynthesis have been divided into an upstream shikimate module containing *aroF^fbr^*, *aroE*, *ppsA*, and *tktA* and a downstream shikimate module with *pheA^fbr^*, *aroA*, *tyrB*, and *aroL*. When the promoters Plac and Ptac were used to regulate the upstream and downstream genes in *C. glutamicum*, respectively, the L-Phe generation was improved to 6.33 g/L. Additional integration of major myoinositol transporter IolT-polyphosphate glucokinase tandem genes *iolT2*-*ppgk* at the *ptsI* locus and deletion of the L-Phe transporter gene *aroP*, lactate dehydrogenase gene *ldhA*, and pyruvate dehydrogenase complex E1p subunit gene *aceE* improved L-Phe production to 9.14 g/L. When fed-batch cultivation was utilized in this strain, production was further enhanced to 15.76 g/L [51]. The *ptsH* involved in the PTS was knocked out, and *galp* and *glk*, which are involved in glucose uptake, were co-expressed to increase PEP supply. Introducing the T495I mutation into TyrR’s DNA binding domain notably enhanced the binding of *aroL* and *mtr* genes, strengthening the synthetic pathway and minimizing by-products. Proteomics analysis suggested that the third step of the SHIK pathway posed a production bottleneck for L-Phe. Therefore, by introducing an engineered *aroD* gene into the *E. coli* Xllp08 strain, the production of L-Phe was significantly enhanced, reaching a concentration of 72.9 g/L. This achievement corresponded to a yield of 0.26 g/g glucose and a productivity of 1.4 g/L per hour in a 5 L fermentor under non-optimized fermentation conditions [52].

To capitalize on the availability of low-cost starting materials, particularly benzyl alcohol, Nie et al. adopted a modular engineering strategy to develop an artificial bioconversion pathway for the synthesis of L-Phe. The initial module was constructed by integrating two highly efficient enzymes: natural threonine aldolase and dehydratase, along with overexpressing the gene *ridA*, significantly enhancing the production of phenylpyruvate, a pivotal precursor for L-Phe synthesis. Subsequently, in the second module, the PheDH and FDHV120S enzymes were co-expressed, catalyzing the conversion of phenylpyruvate into L-Phe. Remarkably, this entire pathway, originating from the low-cost aromatic precursor benzyl alcohol, achieved the production of L-Phe, reported for the first time as cofactor self-sufficient and without additional reductant [128]. This achievement suggests that designing and constructing systematic bioconversion processes have the potential to serve as a viable bio-based strategy for the production of L-Phe from inexpensive precursors.

Route-specific and global regulators have also been engineered in *E. coli* W3110 for L-Phe generation. The L-Phe biosynthesis pathway and intracellular precursors were enhanced by knocking in *aroKB*, *aroG^fbr^*, and *tktA*. The dual transcriptional regulator was removed by deleting *tyrR*. Moreover, the metabolic activity during the stationary phase was increased through the expression of the global regulator Fis gene (*fis*) from a plasmid. The final strain, WF123456+*fis*, achieved L-Phe production at 900 mg/L [53].

### 4.5. Adaptive Laboratory Evolution for Enhanced L-Phe Production

The biosynthesis of amino acid products in *E. coli* poses a significant challenge due to the bacterium’s intrinsic intolerance to high concentrations of products. To address this limitation and enhance the tolerance of industrial strains to inhibitory substrates, an adaptive evolution strategy has been devised. This approach, renowned for its effectiveness, serves as a prevalent method for augmenting stress tolerance, primarily owing to its capacity to effectively generate tolerant phenotypes while maintaining genetic stability [129].

Recently, Wang et al. combined two engineering strategies together, including the reconstruction of the L-Phe biosynthetic pathway and adaptive evolution, resulting in the strain *E. coli* PHE04 with increased production and reduced cell mortality during the final stages of fermentation. Through transcriptional profiling, a transcriptional dual regulator MarA was revealed to improve the cellular tolerance to high concentrations of L-Phe during adaptive evolution. After overexpression of *marA*, the final strain *E. coli* PHE05 achieved L-Phe production at 80.48 g/L and a yield of 0.27 g/g glucose in a 5 L fed-batch fermentation. Notably, this represents the highest titer reported in the literature to date [54].

## 5. L-Valine

**Figure 3:**
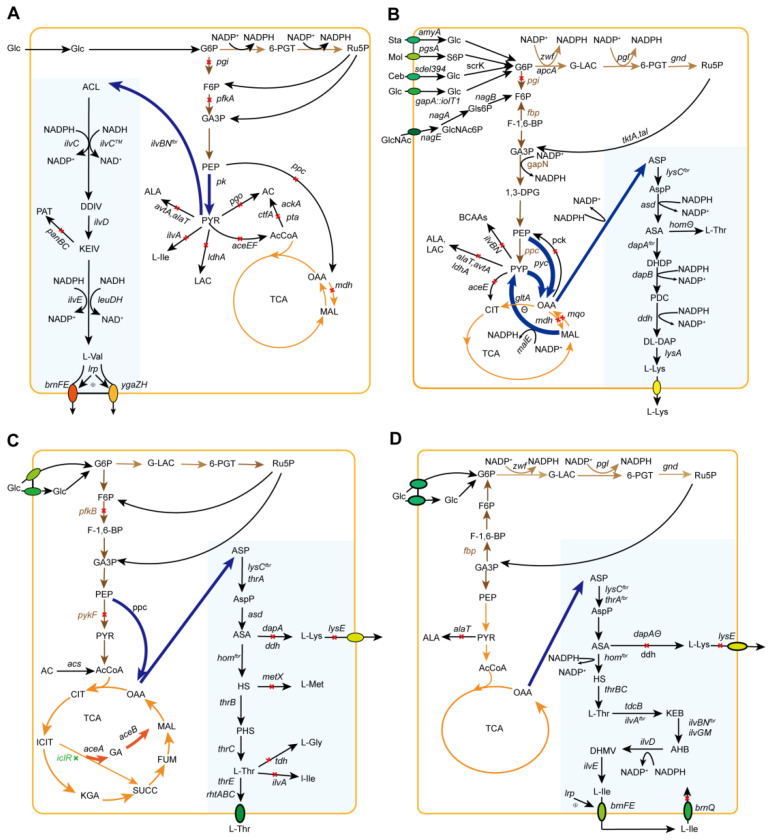
Engineering strategies for enhancing PYR-OAA-node-derived amino acid production. Metabolic flux of (**A**) L-Val, (**B**) L-Lys, (**C**) L-Thr, (**D**) L-Ile. Letters in green indicate an inhibitor of the enzyme. The cross mark indicates the gene is knocked out. The double colon indicates the gene is replaced. The plus and minus symbols indicate activation and repression of the gene expression, respectively. Abbreviations: 6-PGT, 6-phosphogluconate; ACL, 2-acetolatate; AHB, 2-aceto-2-hydroxybutyrate; ASA, L-aspartyl-semialdehyde; ASP, aspartate; AspP, L-aspartyl-phosphate; BCAAs, branched chain amino acids; Ceb, cellobiose; DDIV, 2,3-dihydroxyisovalerate; DHDP, L-2,3-dihydropicolinate; DL-DAP, D,L-diaminopimelate; GA, glyoxylate; GlcNAc, N-acetyl-glucosamine; Gls6P, glucosamine 6-phosphate; HS, homoserine; KEB, 2-ketobutyrate; KEIV, 2-ketoisovalerate; KMV, 2-keto-3-methylvalerate: MAL, malate; Mol, molasses; PAT, pantothenate; PDC, L-piperidine-2,6-discarboxylate; Ru5P, ribulose-5-phosphate; S6P, sucrose-6-phosphate; Sta, starch; *acs*, acetyl-CoA synthetase; *alaT*, alanine transaminase; *amyA*, α-amylase; *asd*, aspartate semialdehyde dehydrogenase; *avtA*, alanine transaminase; *ctfA*, CoA transferase A; *dapA*, dihydrodipicolinate synthase; *dapB*, dihydrodipicolinate reductase; *ddh*, meso-diaminopimelate dehydrogenase; *gapN*, nonphosphorylating NADP-dependent glyceraldehyde 3-phosphate dehydrogenase of *Streptococcus mutans*; *iclR*, isocitrate lyase repressor; *ilvGM*, acetohydroxy acid synthase II; *iolT1*, inositol permease; *leuDH*, leucine dehydrogenase; *lysA*, diaminopimelate decarboxylylase; *lysC*, aspartokinase; *malE*, malic enzyme; *mdh*, malate dehydrogenase; *metX*, homoserine O-acetyltransferase; *mqo*, malate:quinone oxidoreductase; *nagA*, N-acetylglucosamine-6-phosphatedeacetylase; *nagB*, glucosamine-6P deaminase; *panB,* 3-methyl-2-oxobutanoate hydroxymethyltransferase; *panC*, pantoate-β-alanine ligase; *pfkA*, phosphofructokinase; *pfkB*, phosphofructokinase; *pgl*, 6-phosphogluconolactonase; *pgsA*, anchor protein; *pyc*, pyruvate carboxylase; *pk*, pyruvate kinase; *pqo*, pyruvate:quinone oxidoreductase; *scrK*, fructokinase; *sde1394*, β-glucosidase; *tal*, transketolase; *tdcB*, catabolic threonine dehydratase; *tdh*, L-threonine 3-dehydrogenase; *thrA*, aspartate kinase; *thrB*, homoserine kinase; *thrC*, threonine synthase; *thrE*, threonine transporter; *ygaZH*, L-valine exporter.

L-Val is generated mainly through the glycolytic pathway and branched-chain amino acid (BCAA) biosynthetic route (Figure 3A). Glycolysis catabolizes glucose to PYR. L-Val is generated from PYR via four continuous enzymes, including acetohydroxy acid synthase (AHAS), acetohydroxy acid isomeroreductase (AHAIR), dihydroxy acid dehydratase (DHAD), and BCAA transaminotransferase (AT). These enzymes also metabolize the formation of L-Ile originated in PYR and 2-ketobutyrate, which is generated from L-Thr using threonine dehydratase (TD). Ketoisovalerate, the final intermediate of the L-Val formation pathway, is branched to L-leucine (L-Leu) and pantothenate biosynthesis by 2-isopropylmalmate synthase and 3-methyl-2-oxobutanoate hydroxymethyltransferase, respectively. AHAS is the rate-limiting enzyme of BCAA biosynthesis, which is prone to allosteric inhibition by all BCAAs in *C. glutamicum* [130]. Intracellular L-Val is secreted outside the cell through the transport protein ygaZH encoded by *ygaZH*. YgaZH also transports the other two BCAAs L-Ile and L-Leu. The expression and activity of ygaZH have been controlled via the regulatory factor Lrp encoded by *lrp* [131]. The theoretical output is 1 mol of L-Val for every mole of glucose. Producing 1 mol of L-Val from PYR necessitates 2 moles of NADPH. Nevertheless, 1 mol glucose generates 2 mol NADH in the process of PYR production, resulting in surplus NADH and insufficient NADPH for L-Val biosynthesis [132].

It is estimated that approximately 500 metric tons of L-Val has been generated annually [133]. L-Val has been commonly produced by microbial fermentation using random mutation or molecular modification of strains of *C. glutamicum* and *E. coli*. At present, the metabolic engineering strategy for the production of L-Val by microorganisms shares similarities with that of the above three amino acids, mainly including the following: (1) modification of core biosynthetic pathways, (2) elimination of competitive pathways as well as by-products, (3) cofactor engineering, (4) transporter engineering, and (5) optimization of carbon flux. Subsequently, the research progress in metabolic engineering production of L-Val is described.

### 5.1. Engineering to Strengthen L-Val Biosynthetic Pathways and Eliminate Competition Pathways

Enhancing L-Val production by *C. glutamicum* involves the overexpression of critical enzymes involved in the L-Val biosynthetic route and the deletion of core enzymes used in competition pathways. The allosteric inhibition of AHAS was relieved by mutation of the small AHAS subunit *ilvN*. The L-Ile competition pathway was blocked by deleting the TD gene *ilvA*, and the pantothenate branched route was removed through disruption of the ketopantoate hydroxymethyl transferase gene *panB*. Further overexpression of the feedback-resistant AHAS gene *ilvBN^fbr^* and the AHAIR gene *ilvC* was produced in the mutant strain *C. glutamicumΔilvAΔpanB ilvN*M13. The final strain synthesized 15.2 g/L L-Val after 48 h with a yield of 0.38 g/g glucose [55]. In turn, the wild-type strain *Brevibacterium flavum* ATCC14067 was constructed to express *ilvEBN^fbr^C* from the L-Val producer *B. flavum* NV128 to channel additional carbon flux for L-Val production. When the fermentation temperature was increased to 37 °C, the strain produced 38.08 g/L L-Val within 48 h at a yield of 0.241 g/g glucose, with a productivity of 0.133 g/g per hour [134]. Deleting the *ilvA* gene encoding TD decreased the level of 2-ketobutyrate, which resulted in the elimination of the L-Ile competition pathway and switched the AHAS affinity to PYR. The additional deletion of *panBC* encoding ketopantoate hydroxymethyl transferase and pantothenate synthetase inhibited the pantothenate branched pathway and decreased PYR branched flow via PYR dehydrogenase. When the L-Val biosynthetic pathway gene *ilvBNCD* was overexpressed in the double-mutant strain *C. glutamicum* ATCC13032 (*ΔilvAΔpanBC*), L-Val production was improved to 91 mM [135]. Strain *C. glutamicum ilvN*M13, a *panB* mutant, was constructed to amplify *ilvD* and *ilvE* using the strong promoters P*_ilvD_*_M7_ and P*_ilvE_*_M6_ and provide slight expression of *ilvA* by the weak mutant promoter P*_ilvA_*_M1CG_. In this strain, L-Val production reached 136 mM within 48 h by flask cultivation [136]. With the help of a recently developed CRISPR-Cas9 toolkit [137], the AHAS regulatory subunit gene *ilvH* was mutated to reduce L-Val feedback inhibition; *ilvBHC*, *ilvD*, BCAA aminotransferase gene *ybge*, and *ywaA* were overexpressed chromosomally to enhance biosynthetic pathway flux; and the BCAA dehydrogenase gene *bcd* was mutated to block L-Val degradation. The PYR dehydrogenase complex E1α subunit gene *pdhA* was mutated to increase PYR availability, further increasing the L-Val titer. Moreover, the genes *leuA* and *ilvA* were mutated to inactivate the L-leucine and L-isoleucine biosynthesis pathways, respectively. The final strain, *Bacillus subtilis* AW015-5, produced L-Val with a titer of 4.61 g/L in shake flask cultures [138]. In addition, the transcription attenuation *ilvBN*^fbr^ was removed in *E. coli* K12 by the positive feedback module. The regulatory region of the *ilvBN*^fbr^ was replaced by the *ilvC* promoter P*_ilvY_* and *ilvY*, which encodes transcription activator IlvY; this increased L-valine production by 41.9% with a titer of 6.1 g/L in shake flask culture [139].

Promoters function as the pivotal regulators of gene transcription, playing a crucial role in modulating gene expression and subsequent metabolite production in amino acid biosynthesis pathways. However, the current methodologies for identifying strong constitutive promoters in *E. coli* or other microorganisms remain constrained. Recently, Pan et al. introduced an innovative screening approach for identifying strong constitutive promoters in *E. coli*, aiming to enhance the production of L-Val. Their strategy primarily relied on random genomic interruption for constructing promoter libraries coupled with fluorescence-activated cell sorting technology for screening. The identified promoters were subsequently harnessed to refine gene expression and redirect the metabolic flux towards L-Val production. Consequently, a strain exhibiting enhanced L-Val synthesis and production was successfully isolated [140].

### 5.2. Combined Regulation of the POP Node and Cofactor Supply

PYR is a critical chemical in L-Val production as a starting material in the POP. Enzymes linked to PYR formation are H^+^-ATPase, PYR dehydrogenase complex, PEP carboxylase, and PYR: quinone oxidoreductase. The *atpG* gene, which is involved in sugar metabolism, was disrupted to improve PYR synthesis. Further overexpression of a C-terminal truncation of the AHAS gene *ilvBN* and the AHAIR gene *ilvC* in the mutant strain *C. glutamicum* A-1 [*atpG*(S273P)] increased L-Val production to 8.2 g/L [141]. Additionally, *C. glutamicum* ATCC13032(pJC4ilvBNCE) was constructed to decrease the expression level of *aceE* via promoter exchange. The PEP carboxylase gene *ppc* and the PYR: quinone oxidoreductase gene *pqo* involved in the refilling reaction and acetate pathway were knocked out. The resultant strain synthesized 86.5 g/L L-Val with a yield of 0.36 mol/mol and a productivity of 1.6 g/L per hour [56]. In another study, precursor PYR was enhanced by replacing the native promoters of the *aceE* and *gltA* genes with a growth-regulated promoter P_CP_2836_ in the L-Val producer *C. glutamicum* AN02 to inhibit carbon flux from PYR or oxaloacetate to citrate. The engineered strains increased the L-Val titer by 23.9 and 27.3% and the L-Val yield by 43.8 and 62.5%, respectively [142].

The influence of precursor PYR and cofactor NADPH on L-Val generation has been discussed by Blombach et al. [143]. The PYR: quinone oxidoreductase gene *pqo* was deleted in an engineered strain *C. glutamicum* (*ΔaceE*)/pJC4ilvBNCE to redirect carbon flow for PYR accumulation. The knockout of the phosphoglucose isomerase gene *pgi* in this strain to redirect metabolism flow from a central glycolytic pathway toward the PPP rendered more NADPH available for L-Val formation. The strain produced 48 g/L L-Val at a yield of 0.56 g/g glucose in fed-batch fermentation [143]. In another study, the cofactor requirement was adjusted by converting the coenzyme for L-Val production from NADPH to NADH. The strain *C. glutamicum* R (*ΔLDH*) was constructed to express *ilvBN^fbr^*, the NAD-preferring mutant *ilvC^TM^*, *ilvD*, and leucine dehydrogenase gene *LeuDH* from *Lysinibacillus sphaericus* to induce metabolism flow from PYR to L-Val. This strain has achieved a production of 1940 mM L-Val, attaining a yield of 0.63 mol/mol glucose, which currently stands as the highest reported titer [57].

### 5.3. Engineering to Eliminate By-Product Formation

A screened *B. flavum* JV16 strain produced L-Val at a level of 22 g/L with L-Ala as a by-product. The deletion of *avtA* encoding alanine transaminase in the strain inhibited L-Ala formation and improved L-Val production to 27.25 g/L [144]. Strain *C. glutamicum* ATCC13869 (*ΔaceE*), with the deletion of *ilvA* and the alanine transaminase gene *alaT*, increased L-Val production by 44-fold. Additional expression of *ilvBNC_1_*_,_ *lrp_1_*, and *brnFE* improved L-Val production to 51 g/L in fed-batch cultivation with no L-Ala and L-Ile by-products detected [145]. Another genetically engineered strain, *C. glutamicum* BN^GE^C^TM^DLD/*ΔLDH*, produced L-Val at a yield of 0.63 mol/mol, with succinate, L-Ala, and acetate as by-products under oxygen deprivation [57]. Several metabolic strategies were reused in this strain for L-Val production. The acetate route was removed through the inactivation of the phosphotransacetylase gene *pta*, acetate kinase gene *ackA*, and CoA transferase gene *ctfA*. Inserting AHAS^fbr^ and the NAD-preferring mutant AHAIR in the genome led to higher L-Val yield and productivity. The additional deletion of *ppc* and *avtA* suppressed succinate and L-Ala formation, respectively. The final strain produced 1280 mM L-Val in 24 h at a yield of 0.88 mol/mol (near theoretical yield) [146]. In addition, with the individual disruption of *leu* or *ilvA* in *Bacillus licheniformis* WX-02, L-Val production increased by 22 and 14 times with a titer of 33.2 and 21.1 mmol/L, respectively. Further disruption of *ldh* in WX-02*∆leuA* improved L-Val productivity to 0.47 mmol/L per hour [147].

### 5.4. L-Val Biosynthetic Pathway with Transporter Engineering Enhancement and the Carbon Flux Loss Decrement

*E. coli* W, which exhibits excellent physiological properties such as low acetic acid accumulation and high L-Val resistance, was deemed a novel host strain for the improved generation of L-Val by genetic engineering. The genes *ilvA*, encoding a protein that functions in the L-Ile formation route, and *lacI*, which encodes lac repressor, were disrupted with the aim of decreasing the carbon flux of POP node loss to increase L-Val formation and allowing constitutive upregulation of genes under the control of P_tac_ or P_trc_. The regulatory factor Lrp, L-Val transporter *ygaZH*, and L-Val biosynthesis factor *ilvBN^fbr^CED* were co-expressed on a plasmid. The final constructed *E. coli* W (*ΔilvA*, *ΔlacI*) [pTrc184ygaZHlrp, pKBRilvBN^fbr^CED] produced L-Val at 60.7 g/L within 29.5 h at a productivity of 2.06 g/L/h [58].

A constructed *E. coli* Val/pKKilvBN was reengineered to improve L-Val production further. Five enzymes were engineered: AHAIR, TA, and DHAD in the L-Val biosynthesis pathway, along with Lrp and YgaZH, which participate in L-Val export. The recombinant strain achieved an L-Val yield of 0.152 g/g glucose. Based on in silico gene knockout simulation, the PYR dehydrogenase gene *aceF*, 6-phosphofructokinase-1 gene *pfkA*, and malate dehydrogenase gene *mdh* were disrupted to decrease carbon flux loss in the POP node. The final strain produced 7.55 g/L L-Val at a high yield of 0.378 g/g glucose [148]. With the help of metabolic engineering to guide evolution, the double-gene disrupted mutant evolved to grow on glucose as substrate with rates up to 0.31 h^−1^. The genome sequencing of this strain revealed that the G407S mutation in isocitrate dehydrogenase (ICD) led to diminished ICD activity and activated the glyoxylate shunt replenishing OAA required for growth. Upon further introduction of a recombinant plasmid pJC4ilvBNCE into the mutant *C. glutamicumΔppcΔpyc*ICD^G407S^, L-Val production increased to 8.9 g/L at a yield of 0.22 g/g glucose [59].

To achieve the efficient production of L-Val, Hao et al. first screened an L-Val-producing chassis strain *E. coli* VAL03 by ARTP mutagenesis combined with high-throughput screening [149]. Then, the carbon flux for L-Val production was increased by the P*_trc_* promoter replacement, and the production of L-Val was enhanced by the deletion of transporter *brnQ* combined with overexpression of *brnFE* from *C. glutamicum*. In addition, the transcription factor PdhR associated with L-Val synthesis and RpoS were overexpressed and inhibited, respectively. Finally, the engineered strain VAL38 produced 92 g/L L-Val in a 5 L bioreactor with a yield of 0.34 g/g glucose. With the help of a biosensor-driven adaptive evolution strategy by imposing an artificial selective pressure on the fluorescent output of the transcription factor Lrp-based biosensor using fluorescence-activated cell sorting, the evolved strain *C. glutamicum ΔaceE* exhibited a significantly higher growth rate, increased L-Val titers (25%), and a 3–4-fold reduction in by-product formation [150]. Genome sequencing, coupled with the introduction of chosen single-nucleotide polymorphisms into the parental strain, led to the discovery of unprecedented mutations. These mutations had a direct impact on the production of either the product (UreD-E188*, a urease accessory protein) or the by-product (GlxR-T93S, a global regulator). However, the limited understanding of bacterial physiology hampers the identification of alternative and unconventional targets, which holds the potential to enhance the production of strains designed through rational engineering.

## 6. L-Lysine

With oxaloacetate (OAA) as the starting material, the biosynthetic route of L-Lys comprises two parts: the aspartate route and the diaminopimelate (DAP) route (Figure 3B). The aspartate route includes three reactions that are metabolized by aspartate aminotransferase (AAT), aspartokinase (AK), and aspartate semialdehyde dehydrogenase (ASADH), respectively. In addition, the aspartate route is shared for the generation of L-Thr, L-Met, and L-Ile. The DAP route consists of four steps. First, dihydrodipicolinatelinate synthase (DHDPS) condenses PYR and L-aspartate semialdehyde (ASA) into L-2,3 dihydrodipicolinate (DHAP). DHAP is further catalyzed by dihydrodipicolinate reductase (DHDPR) to form L-piperidine-2,6-dicarboxylate (PDC). PDC is then catalyzed to D,L-diaminopimelate (D,L-DAP) using either diaminopimelate dehydrogenase (DDH) on ammonium excess in *C. glutamicum* or the tetrahydrodipicolinate succinylase route under the conditions of low ammonium in *E. coli*. The last step is performed by diaminopimelate decarboxylase (DDC) to yield L-Lys [151]. Four moles of reduced-form NADPH are needed to produce 1 mol L-Lys from OAA. Critical enzymes of the L-Lys formation route comprise AK and DHDPS. AK from *C. glutamicum* is concertedly inhibited by L-Lys plus L-Thr. In contrast, AKI, AKII, and AKII from *E. coli* are inhibited by L-Thr, L-Met, and L-Lys, respectively. DHDPS, which condenses PYR with ASA, competes with HD for the latter precursor. In addition, the activity of DHDPS is feedback-inhibited by L-Lys [61].

At present, the strategy for researchers to synthesize L-Lys by metabolic engineering primarily encompasses four aspects: (1) modification of core biosynthetic pathways, (2) elimination of by-products, (3) cofactor engineering, (4) engineering of strain and its multiple carbon catabolism, and (5) establishment of an artificial rare cryptosystem for mutating and screening high-yielding strains. According to the fourth strategy, L-Lys is regularly generated by fermenting mutant strains of bacteria, such as *Corynebacterium* sp. and *Escherichia* sp. Currently, approximately 2,200,000 tons of L-Lys per year is produced primarily through this method. Once the genome sequences of these strains became available, traditional producers were able to achieve high final titers (80–120 g/L L-Lys) and high carbon yields (40–50%), albeit with low productivity (2.1 g/L/h) due to the detrimental side effects of multiple mutations. However, in a recent study, Becker et al. engineered a genetically defined strain, *C. glutamicum* LYS-12, that synthesized 120 g/L L-Lys at a productivity of 4 g/L per h [62]. In the following subsections, the research progress in the production of L-Lys by metabolic engineering is reviewed.

### 6.1. Engineering the L-Lys Biosynthetic Route for Enhanced L-Lys Generation

As AK and DHDPS metabolize the critical steps in L-Lys production, optimization efforts of the L-Lys generation route have concentrated on these enzymes. Viswanath et al. introduced a plasmid with the feedback-resistant AK (S79V) gene *lysC* into wild-type *C. glutamicum* ATCC13032; consequently, L-Lys production was enhanced by 10.5%, reaching 44.2 g/L [152]. Wang et al. introduced an evolved chimeric AK using a synthetic RNA device into the recombinant *E. coli* WMR [153]. The mutant AK BT3 showed complete resistance to L-Lys and L-Thr inhibition and exhibited 160% enhanced in vitro activity compared to the natural enzyme. Consequently, L-Lys generation was improved to 674 mg/L in batch cultivation. A recombinant plasmid pJC23, including the DHDPS gene *dapA*, was introduced into the excellent L-Lys-producing strain *C. glutamicum* MH20-22B; consequently, L-Lys generation was enhanced from 220 to 270 mM [154]. Zhou and Zeng developed an L-Lys riboswitch for L-Lys synthesis in *C. glutamicum* [155]. The integration of the *lysC* riboswitch from *E. coli* into the chromosome of the L-Lys-producing strain *C. glutamicum* LP917 repressed *gltA* gene expression by the endogenous L-Lys. The engineered strain, LPECRS, produced L-Lys in a yield of 0.23 mol/mol glucose in a shake flask. Additionally, Kind et al. developed a pathway coupling strategy for L-Lys production [156]. Succinyl-CoA from the tricarboxylic acid cycle was channeled into the succinylase branch of the L-Lys pathway in *C. glutamicum* BS87 by disrupting the succinyl-CoA synthetase gene *sucCD*. The mutant BS87 ∆*sucCD* increased L-Lys production by 60%.

In addition, a screened *E. coli* LATR11 produced L-Lys at a titer of 17.6 g/L, with feedback-resistant AKIII (encoded by *lysC*^T344M^) and DHDPS (encoded by *dapA*^H56K^). The L-Lys biosynthetic pathway was enhanced by overexpressing *ppc*, *lysC*^T344M^, the ASADH gene *asd*, *dapA*^H56K^, the DHDPR gene *dapB*, and the DDC gene *lysA* integrated with heterologous expression of the *C. glutamicum* DDH gene *ddh.* The engineered bacteria synthesized 37.2 g/L L-Lys at a productivity of 1.16 g/L per hour in shake flasks. When fed-batch cultivation was used for this strain, production improved to 125.6 g/L at a yield of 0.59 g/g glucose (near theoretical yield) and a productivity of 3.14 g/L per hour [157]. Apart from *E. coli* and *C. glutamicum*, *Bacillus methanolicus* can be used as the host for L-lys production. Overexpression of feedback-resistant gene *hom-1*^D120N,V397F^ in the L-Lys producer *B. methanolicus* M168-20 produced L-Lys at a titer of 11 g/L [158].

### 6.2. Engineering to Regulate the POP Node and Block By-Product Formation

OAA is the critical starting material for L-Lys production. The set of enzymes at the POP node is engineered to enhance the supplementation of OAA pools and decrease its metabolization in the TCA cycle or the reductive arm of TCA for L-Lys generation. Although improving the activity of PEPC and the availability of PYR can enhance supplementation of the OAA pools, PYR also represents a key starting material for synthesizing other products, such as L-alanine, BCAAs, acetyl-CoA, L-lactate, and L-acetate. Disruption of *aceE*, encoding the E1p subunit of the PYR dehydrogenase complex, in the L-Lys-producing *C. glutamicum* DM1729 inhibited L-acetate generation and enhanced L-Lys yield by 40%. Further expression of *ddh*, encoding DDH, and *pyc* improved L-Lys production by 57% and 17%, respectively [151]. A decrease in the AHAS activity in DM1729 by truncating the *ilvN* gene, encoding the small subunit of AHAS, increased L-Lys production by 43%. Complete removal of the activity of AHAS in strain DM1729 by deletion of the *ilvB* gene, encoding the large subunit of AHAS, further improved L-Lys production to 70 mM at a yield of 0.18 mol/mol in batch cultivation [159]. Overexpression of the *pyc* gene in the L-Lys producer *C. glutamicum* DG52-5 led to approximately 50% higher L-Lys accumulation with a level of 50 mM [160]. Similarly, overexpression of *pycA* encoding pyruvate carboxylase and *birA* encoding biotin ligase in an L-Lys *C. glutamicum* AHP-3 via promoter exchange enhanced precursor OAA from PYR and increased L-Lys yield by 55% [161]. Kortmann et al. screened feedback-resistant PC_x_^T132A^ and PC_x_^T343A^ by fluorescence-activated cell sorting based on the L-Lys biosensor pSenLys-Spc [162]. Overexpression of PC_x_^T132A^ and PC_x_^T343A^ from plasmid and chromosome integration in *C. glutamicum* DM1858 ∆*pyc* increased L-Lys production by 19% and 15%, respectively. A point mutation (N917G) was inserted into the *ppc* gene of the L-Lys-producing strain *C. glutamicum* LC298 to release the feedback inhibition of PEPC by aspartate and malate. The mutant strain LP97 increased L-Lys yield by 37% in 1.5 L bioreactor batch culture [163]. The deletion of the PYK gene *pyk* resulted in a mutant *C. glutamicum* that synthesized much PEP. The introduction of a D299N mutation in *ppc,* causing PEPC desensitization, and an S252C mutation in *gltA,* rendering citrate synthase (CS) defective, into the mutant to enhance the precursor OAA supply produced a final strain DRL2 that yielded L-Lys 15.7 g/L, with a productivity 0.56 g/L per hour [63]. In addition, isocitrate dehydrogenase gene *icd* expression was reduced via start codon exchange in the L-Lys-producing strain *C. glutamicum* BS87 to redirect the carbon flux from the TCA cycle to anaplerosis. The engineered strain increased L-Lys yield by 42% in batch cultivation [164].

In addition, a series of mutant strains was developed to assess the effect of different citrate synthase (CS) activity levels on L-Lys production. When the activity of CS decreased to 10% of the original activity via promoter exchange, the engineered strain *C. glutamicum* JVO2-C7 produced L-Lys at a yield of 0.31 mol/mol [154]. However, CS activity changes affect cell growth [165,166]. Therefore, properly adjusting CS activity to balance the cell growth and precursor supply is the wisest choice for increasing L-lysine yield and productivity. To accomplish this, the *ppc* and *pyc* genes from the POP node were inserted into the *pck* and *odx* loci, while the P1 promoter of the TCA cycle’s *gltA* gene was deleted. Additionally, the native promoter of the glutamate dehydrogenase-coding gene *gdh* was substituted with the *Ptac-M* promoter. The resulting strain, *C. glutamicum* JL-69P*_tac-M_ gdh*, was able to produce 181.5 g/L of L-lysine, achieving a productivity of 3.78 g/L per hour in fed-batch culture [64]. Disruption of the *mqo* gene encoding malate:quinone oxidoreductase was performed in a genetically defined L-Lys producer *C. glutamicum* AP-3, with the aim of downregulating the flux of the Krebs cycle and redirecting OAA into L-Lys generation. The engineered strain improved L-Lys yield by 18% at a final level of 15 g/L [167]. Similarly, the promoter of succinyl-CoA synthetase gene *sucCD* was replaced by weak promoter *PdapA* to weaken TCA carbon flux, and L-Lys production increased by 20% compared with the control strain *C. glutamicum* LYS-1 [168]. Disruption of the E1p enzyme gene *aceE* and mutation of the PYR carboxylase gene *pyc* (C1372T and G1A) in *C. glutamicum* generated a mutant strain that accumulated abundant PYR and OAA, respectively. The additional deletion of aminotransferase genes *alaT* and *avtA*, lactate dehydrogenase gene *ldhA*, malate dehydrogenase gene *mdh*, and PEPC gene *pck* and the integrated amplification of *lysC*^C932T^ in combination with exogenous amplification of the *E. coli pntAB* gene in the mutant were performed to remove by-products L-alanine, L-lactate, and succinate and further accumulate precursors PYR and OAA. The final strain, Lys9, produced 526 mM at a yield of 0.42 mol/mol and a productivity of 2.69 g/L per hour using fed-batch fermentation [65].

### 6.3. Engineering to Improve Cofactor NADPH Provision for L-Lys Generation

As 1 mol of L-Lys biosynthesis requires 4 mol of NADPH, the supply of reducing power, NADPH, constitutes an essential cofactor for efficient L-Lys biosynthesis. NADPH is formed primarily via the oxidative PPP. Engineering the PPP and redirecting metabolic flow from glycolysis to the PPP resulted in a marked enhancement in L-Lys generation. Marx et al. deleted the phosphoglucose isomerase gene *pgi* in the strain *C. glutamicum* DSM 5715; consequently, L-Lys generation was enhanced by 51% up to 7.19 g/L [169]. Becker et al. substituted the native promoter of *fbp* encoding fructose 1,6-bisphosphatase (FBP) with the stronger P_EFTU_ in the mutant *C. glutamicum* lysC^fbr^, which increased L-Lys yield from 84.7 to 120.6 mmol/mol glucose [170]. Becker et al. subsequently introduced the mutation A243T into the *zwf* gene encoding G6PD of the L-Lys producer *C. glutamicum* ATCC13032 *lysC*^fbr^ and substituted the endogenous promoters of *zwf* and *fbp* with the stronger P_SOD_ [66]. The point mutation led to a higher affinity of G6PD to NADP^+^ and decreased feedback inhibition by ATP, PEP, and FBP. Finally, L-Lys yield was enhanced from 78.8 to 133.5 mmol/mol sucrose. Ohnishi et al. introduced the mutation S361F into the *gnd* gene encoding 6-phosphogluconate dehydrogenase (6PGD) in the L-Lys-producing strain *C. glutamicum* AHP-3 because the mutant enzyme was less sensitive than the native enzyme to feedback inhibition by FBP, ATP, and NADPH [171]. Consequently, L-Lys generation was enhanced by 15% up to 9.1 g/L.

However, although engineering PPP has a positive effect, relying on this is disadvantageous in product yield because the route inevitably releases 1 mol of CO_2_ accompanied by the metabolism of 1 mol of hexose [172]. To address this issue, Takeno et al. substituted the natural NAD-dependent glyceraldehyde 3-phosphate dehydrogenase (GAPDH, *gapA* gene product) with nonphosphorylating NADP-dependent GAPDH (GapN, *gapN* gene product) from *Streptococcus mutans* and then spontaneously introduced the mutation R696C in *rho*, encoding the transcription termination factor Rho, in the mutant ATCC13032 (*ΔgapB*)/pCAK311 [173]. Consequently, L-Lys generation was enhanced by 0.9- and 1.2-fold, reaching 46.7 mM and 0.28 mol/mol fructose, respectively, although the strain still showed retarded growth. For integrating promoted cell growth with a high-level L-Lys synthesis, the chromosomal *iolT1* gene of this strain was substituted with the *gapA* gene under the native *iolT1* gene promoter, which is induced by myo-inositol even in the presence of glucose. With seed inoculum containing 1.5% glucose plus 0.5% myo-inositol, the constructed strain RE2A^iol^/pCAK311 produced L-Lys at 63.5 mM, with a productivity of 2.04 mM/h [67]. Similarly, the substitution of native NAD^+^-dependent GAPDH and NADP^+^-dependent isocitrate dehydrogenase with NADP^+^-GAPDH and NAD^+^-isocitrate dehydrogenase from *C. acetobutylicum* and *S. mutans* in an L-Lys-producing strain *C. glutamicum* JL-6, respectively, balanced the intercellular redox state, and the resulting mutant strain RG1 improved L-Lys significantly to a titer of 121.4 g/L, with a productivity of 0.53 g/g per hour and yield of 0.46 g/g glucose in fed-batch fermentation [174]. Alternatively, expression of the *E. coli pntAB* gene encoding membrane-integral nicotinamide nucleotide transhydrogenase in the L-Lys-producing strain *C. glutamicum* DM1730 reduced the NADP^+^ to NADPH, and the resulting recombinant strain increased the L-Lys titer by 300% using sucrose as carbon source after 72 h of fermentation [175]. In addition, replacing NADPH-dependent dehydrogenases with NADH-utilizing counterparts in the L-Lys biosynthesis pathway is an alternative strategy for L-Lys production. Individual overexpression of *adh* encoding aspartate dehydrogenase from *P. aeruginosa*, *asd* encoding aspartate-semialdehyde dehydrogenase from *T. mobilis*, *dapB* encoding dihydrodipicolinate reductase from *E. coli*, and *ddh* encoding diaminopimelate dehydrogenase from *P. thermarum* in the basic L-Lys-producing strain *C. glutamicum* LC298 increased L-Lys production by 30.7, 32.4%, 17.4%, and 36.8%, respectively. When the native *aspC*, *asd*, and *dapB* were replaced by the *adh_Pa*, *asd_Tm*, and *dapB_Ec* via homologous recombination in the L-Lys hyperproducer *C. glutamicum* ATCC21543, L-Lys production was increased by 30.7% at a titer of 27.7 g/L with a yield of 0.35 g/g glucose in shake flask culture [176]. Also, replacement of the native *dapB* gene with *Ec*-*dapB*^C115G,G116C^ in strain JL-6 switched the nucleotide-cofactor preference of DHDPR from NADPH to NADH, and the mutant strain produced 117.3 g/L L-Lys, with a productivity of 2.93 g/L per hour and yield of 0.44 g/g glucose in a 5 L bioreactor fed-batch fermentation [68].

### 6.4. Engineering of C. glutamicum for L-Lys Production

Although classical whole-cell mutagenesis and molecular techniques have achieved considerable success, random-mutation-obtained producers are mainly inferior to native strains concerning fermentation performance factors such as cell growth, sugar metabolism, and stress resistance. The L-Lys-producing strain *C. glutamicum* GRLys1 was constructed to express *iolT2* encoding inositol permease from *C. glutamicum* ATCC 13032 and *glcK* encoding glucokinase from *B. subtilis* subsp. *Subtilis* str. 168 to enhance glucose uptake and phosphorylation. The additional deletion of *sugR* encoding global regulator SugR and *ldhA* encoding L-lactate dehydrogenase in the recombinant strain derepressed glycolytic and PTS genes and eliminated by-product L-lactate formation. The constructed strain increased L-Lys specific productivities by 70–72% [69]. Becker et al. systematically modified the metabolism of *C. glutamicum* with several engineering strategies for L-Lys production [62]. The L-Lys biosynthetic flux was increased through an additional copy of *ddh* and *lysA*, overexpression of *lysC* (T311I) and *dapB* under the promoter P_SOD_, and nucleotide exchange in the *hom* (V59A) gene. The precursor supply flux was enhanced through deletion of the PEPC gene *pck*, overexpression of *pyc* (P458S) under the control P_SOD_, and substitution of the start codon ATG with the rare codon GTG in the *icd* gene. The PPP flow was improved by substituting the wild-type promoter of *fbp* and the *tkt* operon with the promoters of P_EFTU_ and P_SOD_, respectively. The final strain, LYS-12, generated 120 g/L L-Lys at a yield of 0.55 g/g glucose and productivity of 4 g/L per hour by fed-batch cultivation [177]. Xu et al. engineered a plasmid-free L-Lys hyper-producing strain, Lys5-8, from the genetically defined L-Lys producer Lys5 [178]. In Lys5-8, *gapA* was replaced with the NADP-dependent GAPDH gene *gapC* from *C. acetobutylicum* to increase glucose consumption and NADPH availability. Disruption of the C-terminal domain in *ilvN* and site-directed mutation of *hom* decreased the by-product accumulation and further enhanced the precursor availability. The *pck*, *mdh*, *alaT*, *ldhA*, *avtA*, and *aceE* fragment was replaced with a *lysC*^C932T^, *asd*, *dapA*, *dapB*, *ddh*, and *lysA* cassette to enhance L-Lys biosynthetic flux. In fed-batch culture, *C. glutamicum* Lys5-8 could synthesize 896 mM L-Lys, at a productivity of 2.73 g/L per hour and glucose conversion rate of 47.06%. In addition, Wang developed a high-throughput screening strategy for L-Lys production. An L-Lys high-producing *E. coli* LYS2 underwent evolution driven by an atmospheric and room temperature plasma mutation system based on the pA-*egfp* biosensor. The mutant strain produced 136.51 g/L in 5 L bioreactor fed-batch fermentation [179].

### 6.5. Engineering to Enhance Multiple Carbon Catabolism in C. glutamicum

A *C. glutamicum* ATCC13032 (*Δhom*) mutant was constructed to express the beta-glucosidase gene *sde1394* from *Saccharophagus degradans* and display the enzyme on its cell surface using the porC anchor protein. The strain catabolized cellobiose to synthesize L-Lys at a titer of 1.08 g/L after four days of cultivation [180]. To enhance L-Lys production, a *C. glutamicum* DM1729 mutant (*lysCP458S*, *homV59A*, *pycT311I*) was engineered to co-express endoglucanase genes from *Xanthomonas campestris* XCC2387 and betaglucosidase gene from *Saccharophagus degradans* Sde1394 on the cell surface, utilizing the porC anchor protein. This strain, when cultured with cellobiose alone or combined with carboxymethyl cellulose, achieved an L-Lys titer of 5.9 mM after 96 h of fermentation [181]. Additionally, the L-Lys-producing strain *E. coli* WC196LC (pCABD2) was developed, expressing the phospho-α-glucosidase gene *glvA* and phosphoenolpyruvate-dependent maltose phosphotransferase system subunit gene *glvC* from *Bacillus subtilis* 168, to facilitate the efficient utilization of isomaltose and panose in industrial glucose feedstocks. The engineered strain increased L-Lys yield by 27.7% at a titer of 7 g/L from maltose, isomaltose, and panose [182]. A screened *C. glutamicum* XQ-8 strain produced L-Lys at a titer of 97.6 g/L, with low glucose consumption. The glucose consumption was enhanced by overexpressing the PTS^GLC^ gene *ptsIH* and disrupting the global repressor gene *sugR*. The constructed strain produced 159.2 g/L L-Lys from glucose plus maltose, with a high productivity of 3.25 g/L per hour in fed-batch fermentation. However, the strain generated large amounts of by-products, including pyruvate, lactate, L-Val, and L-Ala [157]. Alternatively, expression of non-PTS^GLC^ genes *iolT1*, *iolT2,* and *ppgK* via promoter exchange reduced by-product accumulation, and the mutant strain *C. glutamicum* ZL-92 produced 201.6 g/L L-Lys with a productivity of 5.04 g/L per hour and a yield of 0.65 g/g glucose in 5 L bioreactor fed-batch fermentation, which is the highest value reported so far [70]. In addition, the L-Lys transport gene *ybjE* from *E. coli* and feedback-resistant AK gene *lysC^fbr^* were expressed in the *Synechococcus* sp. PCC 7002_A2542 and _A1838 loci under the control of P_clac94_ and P_EZtet_ induction systems, respectively. The mutant produced L-Lys at a titer of 2.7 mM with CO_2_ as the sole carbon source [183]. Malla et al. screened a novel efficient L-Lys transporter MglE by functional metagenomics [184]. The yield, titer, and the specific production of L-Lys in an industrial *C. glutamicum* strain increased by 7.8%, 9.5%, and 12%, respectively. They provided an approach to the discovery of novel exporters that can be deployed to improve titers and productivity of the toxic chemicals produced by fermentation.

The strain *C. glutamicum* (*lysC^fbr^ dld_PSOD_ pys_PSOD_ malE_PSOD_*) was constructed to overexpress the FBP gene *fbp* and GAPDH gene *gapX* by the same P_SOD_. This strain multiplied on grass and corn silages with a specific growth rate of 0.35 h^−1^ and L-Lys yield of roughly 90 C-mmol/C-mol [185]. Upon foreign amplification of *fbp* from *E. coli* and the fructokinase gene *scrK* from *C. acetobutylicum* in *C. glutamicum lysC*^fbr^ with molasses as the sole C source, L-Lys synthesis was increased by 88.4%, reaching 47.1 mM [186]. Foreign expression of the *Corynebacterium glycinophilum* DSM45794 EII permease gene *nagE* in the L-Lys producer *C. glutamicum* DM1729 through start codon exchange (GTG-ATG), as well as the introduction of the 5′-GAAAAGGAGG-3′ ribosomal binding site of the 16S rRNA gene upstream of the *nagE* ATG start codon via PCR, led to N-acetyl-glucosamine (GlcNAc) uptake. Further overexpression of the endogenous N-acetylglucosamine-6-phosphatedeacetylase gene *nagA* and glucosamine-6P deaminase gene *nagB* in the recombinant *C. glutamicum* led to GlcNAc utilization. The resulting strain DM1729 (pVWEx1-*nagE*)(pEKEx3-*nagAB*) produced 25.7 mM L-Lys at a yield of 0.17 g/g GlcNAc [187]. Tateno et al. amplified the α-mylase gene *amyA* from *Streptococcus bovis* 148 and exhibited the enzyme on its cell surface using the PgsA anchor protein from *Bacillus subtilis* [71]. The engineered strain *C. glutamicum Δhom*::HPA metabolized soluble starch to generate 6.04 g/L L-Lys at a yield of 0.19 g/g starch. The mannitol repressor MtlR was deleted, and fructokinase was expressed in the L-Lys-producing strain *C. glutamicum* LYS-12. The engineered strain produced small amounts of L-Lys due to low flux into the PPP, which resulted in a limited NADPH supply. Thus, a strategy of overexpressing the NADP-dependent GapN from Streptococcus mutans and coupling the EMP pathway flux to NADPH formation was used in this strain. When grown on mannitol, the final *C. glutamicum* SEA-3 produced an L-Lys yield of 0.24 mol/mol and a specific productivity of 1.3 mmol/g per hour [188].

### 6.6. Establishment of Artificial Rare Cryptosystem for Mutating and Screening High-Yielding Strains

Targeting the development of biosensors with tailored and versatile responses to intracellular L-Lys holds significant promise for advancing high-throughput screening of L-Lys-producing strains, thereby representing a pivotal area for future research endeavors [189]. Notably, the emergence of high-throughput fluorescence-activated cell sorting (FACS) has garnered considerable attention as an innovative and potent screening platform, enabling the efficient screening of large strain libraries [190].

In a recent study, an *E. coli* mutant strain exhibiting enhanced L-Lys production was successfully isolated through the utilization of a fluorescence-based screening method and an *E. coli* strain deficient in five out of six L-Lys tRNA-UUU genes. This approach initially yielded a screening marker enriched in rare codons, which exhibited a positive correlation with L-lys content. Subsequently, through a combination of constant temperature and atmospheric pressure plasma mutagenesis, along with the induction of fluorescent protein expression, mutant strains exhibiting robust fluorescence were isolated via flow cytometry. Following a 48 h fermentation process, a high-yielding L-Lys strain named *E. coli* QD01 *ΔtRNA* L2 was obtained, demonstrating a remarkable production of 14.8 g/L L-Lys, surpassing the wild-type strain’s production by 12.1% [72]. The screening strategy established in this study provides an efficient, accurate, and easy method for screening product-targeting microorganisms.

## 7. L-Threonine

L-Thr biosynthesis, starting with L-aspartate, involves five enzymatic steps catalyzed by AK, ASADH, HD, homoserine kinase (HK), and threonine synthase (TS) [191]. AK constitutes the first rate-limiting enzyme in the L-Thr route, which helps to push the carbon flow toward the aspartic family amino acids. In *E. coli*, three AK isoenzymes, AKI, AKII, and AKIII (encoded by *thrA*, *metL*, and *lysC*, respectively), are feedback-repressed and/or feedback-inhibited by L-Thr, L-Met, and L-Lys, respectively. AKI and AKII comprise bifunctional enzymes containing two catalytic domains, one with AK activity and the other with HD activity, also known as AKI-HDI and AKII-HDII [192]. In *C. glutamicum*, AK encoded by *lysC* is feedback-inhibited by L-Lys plus L-Thr [193]. In *E. coli* and *C. glutamicum*, AHADH is encoded by *asd*. Its synthesis is repressed only in *E. coli* by L-Lys, L-Thr, and L-Met, and its activity is not influenced by the end product. HD, the second rate-limiting enzyme in the L-Thr route, controls carbon flow toward L-homoserine formation. It competes with DHDPS for ASA to synthesize L-Lys. In *E. coli*, two isoenzymes of HD are present as bifunctional AKI-HDI and AKII-HDII. HDI is susceptible to inhibition by L-Thr. In *C. glutamicum*, HD is encoded by *hom*. Its expression is slightly repressed by L-Met, and its activity is inhibited by the end-product L-Thr. HK comprises the third critical enzyme of the L-Thr route, regulating carbon flow toward L-Thr generation. It competes with homoserine O-acetyltransferase, encoded by *metX*, for homoserine to synthesize L-Met [194]. In both *E. coli* and *C. glutamicum*, HK is encoded by *thrB*, and TS is encoded by *thrC*. In *E. coli*, *thrA*, *thrB*, plus *thrC* constitute the *thr* operon. The expression of the *thr* operon is repressed by L-Thr plus L-Ile, which also inhibits the activity of HK. In *C. glutamicum*, *hom* and *thrB* comprise the *thr* operon. The transcription of *thrB* is slightly repressed by L-Met, and the activity of HK is allosterically inhibited by L-Thr [130].

L-Thr is depleted for the generation of L-Ile and L-glycine (L-Gly). The consumption of L-Thr toward L-Ile is initiated by TD. Both *E. coli* and *C. glutamicum* contain two isozymes of TD encoded by *tdcB* and *ilvA*, respectively. The consumption of L-Thr toward L-Gly in *E. coli* can be catalyzed by two isozymes, L-Thr 3-dehydrogenase encoded by *tdh* and L-Thr aldolase encoded by *ltaE*. In *C. glutamicum*, L-Thr is cleaved directly by Ser hydroxymethyl transferase, encoded by *glyA*, into L-Gly and acetaldehyde [195]. In *E. coli* and *C. glutamicum*, the export of L-Thr mainly occurs through carrier-mediated excretion. Three permeases, RhtA, RhtB, and RhtC, encoded by the three separate genes *rhtA*, *rhtB*, and *rhtC* in *E. coli* can export L-Thr out of the cell, whereas the export of L-Thr in *C. glutamicum* is dependent on the transporter ThrE encoded by *thrE* (Figure 3C) [196].

Currently, the strategy for producing L-Thr via metabolic engineering of *E. coli* and *C. glutamicum* is primarily divided into the following points: (1) modification of the core biosynthetic pathway, (2) elimination of by-products, (3) transport engineering, (4) dynamic regulation of metabolic pathways (e.g., POP node and operon), and (5) system metabolic engineering. Notably, Liang et al. employed system metabolic engineering to significantly enhance the production of L-Thr in *E. coli*, achieving the highest reported yield thus far [73]. The following subsections detail the specific research progress in L-Thr production using these two types of engineered bacteria.

### 7.1. L-Thr Biosynthetic Pathway Modification and By-Product Elimination

As the three key enzymes, AK, HD, and HK, control L-Thr biosynthesis, the engineering of the L-Thr biosynthetic pathway has been focused on these enzymes. A recombinant pJD4 harboring the *hom^fbr^*-*thrB* operon was introduced into the L-Lys producer *Corynebacterium lactofermentum* ATCC21799 [195]. The developed strain could generate 5.4 g/L L-Thr, whereas the L-Lys synthesis was significantly decreased from 22 to 4.5 g/L. However, 2 g/L by-product homoserine was also generated in the medium. When *hom^fbr^* and *thrB* were controlled by their native promoter and the P_tac_ in this strain, respectively, L-Thr production increased to 11.8 g/L. The by-product L-Lys was further decreased to 0.8 g/L, and L-homoserine was not detected in the fermentation broth, although 4.6 g/L L-Ile and 1.9 g/L L-Gly still accumulated. The disruption of genes *ddh* and *lysE* resulted in a recombinant *C. glutamicum* that synthesized little L-Lys (0.42 g/L). The transformation of a recombinant plasmid including *lysC^fbr^*, *hom^fbr^*, and *thrB* into the mutant to enhance the L-Thr carbon flux yielded a resultant strain that synthesized L-Thr at 7.27 g/L [197]. The genes *metX* and *dapA* were first disrupted in *C. glutamicum* R102 to reduce the by-product L-Met and the L-Lys formation. Sequentially, a plasmid-harbored L-Thr operon gene *hom*-*thrB* and exporter gene *thrE* were transformed into the mutant. The engineered strain increased L-Thr production by 86.1%, from 1.8 to 3.5 g/L [74]. Wei et al. developed a promoter-library-based module combination technology in *C. glutamicum* for L-Thr production [75]. Three genes were first disrupted, including *ilvA*, *metX*, and *tdcB*. Further expression of *lys^fbr^*, *hom^fbr^*, *asd*, *thrB*, and *thrC* was controlled by high-, medium-, and low-strength promoters based on plasmid pXMJ22. The L-Thr production of the constructed strain was 12.8 g/L.

In *E. coli*, scaffold plasmid pSC-4, which contains one, one, and two sites for artificial DNA-binding domain (ADB)-fused enzymes (HD-ADB1, HK-ADB2, and TS-ADB3), and the pET16tac-*thrABC* plasmid, which amplified the above three ADB-fused enzymes under the regulation of P_tac_, were introduced into the L-Thr producer *E. coli* MG-105. As a result, L-The production time was reduced by over 50%, and the cell growth rate of this strain was significantly enhanced by decreasing the intracellular accumulation of metabolic intermediates such as homoserine [198]. Three pathways were designed in *E. coli* TWF006 for L-Thr production. The fatty acid degradation and L-Thr biosynthesis were enhanced by deleting *fadR* and *fabR* and inserting *fadBA* into the *lacZ* locus. The glyoxylate shunt was strengthened by disrupting *lacI*, inserting *aceBA* into the *lacZ* locus, and replacing the native promoter of *acs* with *P_tac-trc_* to direct the carbon flux into the POP node. The L-Thr biosynthesis pathway and precursor OAA formation were improved via the insertion of *P_tac_*-*thrA^fbr^BC*-*rhtC* into the *lacA* locus and the integration of *P_tac_*-*aspC* and *P_tac_*-*ppc* into the *lacZ* locus. The resulting strain, TWF044, produced L-Thr under the fed-batch condition at a titer of 103.89 g/L and a yield of 0.72 g/g glucose, with a productivity of 2.16 g/L per hour [76]. Similarly, four pathways were redesigned in *E. coli* MG1655 for L-Thr production. The PTS system was blocked to save PEP by disrupting *crr*, and the TCA cycle was improved by exchanging the *gltA* gene native promoter with P*trc*. The glyoxylate cycle was manipulated by deleting the *iclR* gene, and the L-Thr biosynthesis pathway was strengthened by overexpressing the essential genes *thrA^fbr^BC*-*rhtC* from the plasmid. The final strain, WMZ016/pFW01-thrA*BC-*rhtC*, produced 17.98 g/L L-Thr with a yield of 0.346 g/g glucose [77]. In addition, the deletion of *pfkB* encoding phosphofructokinase and *pykF* encoding PYK in an L-Thr producer of *E. coli* THRD inhibited acetate formation and increased L-Thr yield to 0.37 g/g glucose at a level of 111.4 g/L [78]. The expression of some critical genes responsive to L-Thr was controlled dynamically by the attenuator region of the *thr* operon, *thrL*. Different RBS strengths in *thrR* (*RBS_thrL_*-*thrL*-*RBS_thrA_*) were inserted in the *iclR* regulator region in TWF001. The best strain, TWF063, produced 16.34 g/L L-Thr. With the additional integration of the fragment *P_cysH_*-*RBS_s7_*-*aspC* into the *lacI* loci in TWF063, L-Thr production increased to 17.56 g/L. Upon further repression of the gene *arcA* in the TCA cycle; the genes *fadR*, *cpxR,* and *gadE* in fatty acid synthesis; and the gene *pykF* in aerobic respiration in TWF066 by the dynamic regulation of *thrR*, the L-Thr fermentation titer reached 25.50 g/L. The final strain TWF083 produced L-Thr 116.62 g/L with a yield of 0.486 g/g glucose and productivity of 2.43 g/L per hour in a fed-batch fermentation [79]. Liu et al. developed a novel PS-Brick technology for iterative, seamless, and sequence-repeat DNA assembly [199]. The feedback regulation of L-Thr *thrABC* operon was first released. After that, the metabolic bottleneck for L-Thr synthesis and efflux was identified by further deletion of genes *tdh* and *ilvA* using sgRNA arrays created with the TA clone/BciVI-based PS-Brick strategy. Finally, a recombinant plasmid pACYC184-*thrA^fbr^BC-asd*-P_T_BCD1-*rhtC* was transformed into the mutant *E. coli* 1655*ΔtdhΔilvA*. The constructed strain produced L-Thr at a titer of 45.75 g/L in a 7.5 L fermentor fed-batch fermentation. In another study, the introduction of the poly-3-hydroxybutyrate biosynthesis pathway to *E. coli* strain TWF001, an L-Thr-producing strain, produced L-Thr with a titer of 133.5 g/L after fed-batch culture in the 10 L fermenter. In this study, *aspC* and *thrABC* involved in the L-Thr biosynthesis were upregulated, *poxB*, *pta*, and *ackA* involved in the acetate formation were downregulated, and the *acs* gene encoding acetyl-CoA synthetase was involved in the acetyl-CoA formation from acetate. The expression of the gene cluster *phaCAB* from *Ralstonia eutropha* showed extreme importance in rebalancing metabolic flux distribution for final L-Thr generation [200].

### 7.2. Transport Engineering for Enhanced L-Thr Production

After strengthening the L-Thr biosynthetic pathway and eliminating its competitive branch, the L-Thr export system represents the next engineering target. Overexpression of the L-Thr exporter ThrE gene *thrE* in the L-Thr producer *C. glutamicum* MH20-22B-3 (*hom*^fbr^-*thrB*) enhanced L-Thr synthesis from 5.8 to 8.1 g/L with decreased generation of glycine and L-Ile [195]. Moreover, upon foreign expression of the *rhtC* gene of *E. coli* in the L-Thr producer *C. glutamicum* DM368-3, L-Thr synthesis increased from 0.9 to 3.7 g/L without L-homoserine formation [201].

Kruse et al. introduced the engineered plasmid pTrc99A-*thrE* into the L-Thr producer *E. coli* MG422 [202]. Consequently, the specific L-Thr production rate was improved by 290%. Livshits et al. enhanced *rhtA* expression through a mutation (G→A) one base upstream of the *rhtA* gene start codon in the genome of *E. coli* MG422 (pAYC32-*thrA^fbr^BC*) [80]. Consequently, L-Thr synthesis was enhanced from 18.4 to 36.3 g/L. Additionally, the L-Thr production was manipulated by the regulator. The deletion of *acrA* encoding global regulator ArcA and *iclR* in the L-Thr-producing *E. coli* TWF001 directed numerous fluxes into the TCA cycle and glyoxylate shunting. Further deletion of *tdcC* in the mutant prevented the transport of L-Thr from the outside to inside *E. coli* cells. The final constructed strain, TWF018, increased the L-Thr yield by 109.7% at a titer of 26 g/L [203].

### 7.3. Engineering to Dynamically Regulate the POP Node and Operon

To coordinate cell growth and product formation, dynamically regulating the POP node is becoming an effective metabolic engineering strategy. PYR carboxylase gene *pycA* from *B. subtilis* was heterologously expressed on the low-copy-number plasmid pWSK29 in the L-Thr producer *E. coli* THRD. The constructed strain increased the L-Thr yield by 12.3% at a final titer of 46.10 g/L [60]. Liu et al. utilized a dynamic regulation strategy in the L-Thr-producing strain *E. coli* THRD for L-Thr production [204]. The expression of *pckA* from *M. succiniciproducens*, *pycA* from *L. lactis*, and *citrA* from *A. niger* under the control of constitutive promoter pBBa_J231175 and inducible promoter placUV5-tetO improved growth performance at the beginning of fermentation. At the exponential phase, IPTG was added to switch off the *citA* expression and induce the expression of *aspC*, *fdh* from *C. boidinii*, *gdhA*, and *pntAB* under the control of placUV5-tetO and placUV5 for directing the carbon flux into L-Thr biosynthetic pathway. When sodium formate was supplied in the strain, L-Thr production increased to 70.8 g/L with a yield of 0.404 g/L glucose and productivity of 1.77 g/L per hour. A thermal switch system has recently been applied to *E. coli* for L-Thr maximization. Eight genes were first knocked out, including *poxB*, *pflB*, *ldhA*, *adhE*, *tdcC*, *avtA*, *alaA*, and *alaC*. A recombinant *E. coli* expressed the *rhtC* from *E. coli* MG1655, *pyc* from *Lactococcus lactis*, and *alaA* under the control of a thermosensitive circuit *cI^ts^*-*P_R_*-*P_L_* and a stringent circuit *tetR*-*P_LtetO-1_* from plasmid pFT24. When the fermentation temperature was set at 37 °C at first 6 h and then shifted to 42 °C, the resulting strain TWF113/pFT24rpa1 produced L-Thr at a yield of 124.03% mol/mol, which exceeds the maximum available theoretical value (122.47%) [81]. In other metabolic regulation strategies, Yang et al. added L-glutamic acid into a medium for migrating the metabolic flow to L-Thr, resulting in a titer of 77 g/L by fed-batch fermentation, 10% higher than that without the addition of L-glutamic acid [205].

The *thrABC* operon holds a pivotal role in orchestrating the biosynthesis of L-Thr. Hao et al. conducted a thorough analysis, initially examining the impact of varying proportions of *thrAB* and *thrC* separation on the growth and L-Thr production of *E. coli*. Subsequently, they implemented a stationary phase promoter to dynamically modulate the expression of the engineered *thrABC* operon, achieving enhanced cell growth and a shortened fermentation period. Furthermore, through the deletion of the *ptsG* gene, they reduced acetate metabolic overflow, thereby boosting L-Thr production. The culmination of these strategies resulted in the development of the final strain, P2.1-2901Δ*ptsG*, which attained a remarkable titer of 40.06 g/L after 60 h of fermentation, surpassing all previously reported titers in shake flasks [82].

### 7.4. Systems Metabolic Engineering

A genetically defined *lacI*-mutant strain of W3110 was engineered to produce L-Thr. Feedback inhibition of AKI and AKIII and transcriptional attenuation of the *thr* operon were released, and the deregulated *thr* operon was overexpressed from a plasmid. Routes for L-Thr consumption were released by disrupting *tdh* and mutating *ilvA*. The *metX* and *lysA* genes were also disrupted to leave more carbon flux available for L-Thr formation. Thereafter, further stepwise modifications were carried out based on transcriptome analysis and in silico metabolic simulation. The native promoter of *ppc* was substituted with P_trc_ to strengthen the refilling flow. The *iclR* gene encoding the transcriptional regulator IclR of isocitrate lysase and malate synthase was deleted to increase glyoxylate shunting. The *tdcC* gene encoding an L-Thr importer was knocked out to prevent L-Thr import. The three genes *rhtA*, *rhtB*, and *rhtC* were amplified from a plasmid to promote L-Thr secretion. The wild-type promoter of the *acs* gene encoding acetyl-CoA synthetase was replaced with P_trc_ to decrease acetic acid accumulation. The resultant constructed *E. coli* strain synthesized 82.4 g/L L-Thr, with a productivity of 1.65 g/L per hour and a yield of 0.393 g/g glucose using fed-batch cultivation [206]. In addition, a simplified-chromosome *E. coli* MDS42 was reengineered for L-Thr production. The wild-type *thr* operon was replaced with a feedback-resistant *thrA^fbr^BC* operon under the regulation of P_Tac_, and the *lacI* gene was disrupted. The *tdh* gene was knocked out to prevent the degradation of L-Thr. The *tdcC* and *sstT* genes encoding an L-Thr importer were replaced with a mutant L-Thr transporter gene *rhtA23*, which enhanced L-Thr export and blocked its re-uptake. The resulting strain MDS-205 generated 40.1 g/L L-Thr at a yield of 0.40 g/g glucose within 30 h by batch culture, representing 40% of the theoretical value [83]. Recently, Liang et al. have developed an effortless and practical approach for crafting polyploid *E. coli*, successfully demonstrating its efficacy in enhancing L-Thr production. This artificially induced polyploid strain, harboring 2–4 chromosomes, boasts a larger cellular dimension, along with enhanced tolerance to low pH and acetate stress, surpassing its haploid counterpart. Notably, the genes encoding the cell’s core functional pathways exhibit significant upregulation, contributing to the strain’s remarkable performance. These advancements have culminated in the achievement of the highest L-threonine yield reported thus far, attaining a yield of 160.3 g/L in fed-batch fermentation [73], marking a significant milestone in metabolic engineering.

## 8. L-Isoleucine

L-Ile formation consists of ten enzyme reactions, originating from the metabolism intermediate L-aspartate and branching synthesis of L-Lys and L-Met (Figure 3D). The first five enzymes, AK, ASADH, HD, HK, and TS, metabolize sequentially and convert L-aspartate to L-Thr. L-Thr is further metabolized by TD to generate 2-ketobutyrate. The last four enzymes, AHAS, AHIAR, DHAD, and AT, participate in the formation of L-Val, L-Leu, and pantothenate. The ASD, HD, and AHIAR enzymes require the coenzyme NADPH in the ten metabolic reaction routes. NADPH can be synthesized by two types of reactions. One is the reductive regeneration of NADPH via NADP^+^-dependent dehydrogenases, such as G6PD and 6PGD in the PPP, malic enzyme, and isocitrate dehydrogenase. Another is the phosphorylation of NAD^+^ and NADH via NAD^+^ kinases and NADH kinases, respectively, such as inorganic polyphosphate/ATP-NAD kinase Ppnk and NADH kinase Pos5 [84]. The L-Ile carbon flow regulation is operated by repression of *thrA*, *lysC*, and *ilvGM* (encoding AHASII), along with *hom*-*thrB* and *ilvBNC* operons in *E. coli* and *C. glutamicum*, respectively. The activities of key enzymes AK, HK, HD, and AHAS are controlled by one or more amino acids such as L-Lys, L-Met, L-Thr, L-Ile, and L-Val. Intracellularly produced L-Ile is secreted through the transporter BrnFE which is controlled by the regulatory factor Lrp [85].

The current strategy for producing L-Ile by metabolic engineering is mainly divided into the following four points: (1) modification of core biosynthetic pathways, (2) elimination of competitive pathway, (3) cofactor engineering, and (4) secretion engineering. The following is the specific research progress in the production of L-Ile using *E. coli* and *C. glutamicum*.

### 8.1. Engineering to Enhance L-Ile Biosynthesis and Weaken Competing Branches

Once the genomes of *C. glutamicum* and *E. coli* were sequenced, the biosynthesis route of L-Ile became a primary target for enhancing L-Ile generation. The L-Thr dehydration pathway was enhanced by expressing the catabolic gene *tdcB* of *E. coli* in the L-Lys producer *C. lactofermentum* ATCC21799 to redirect the carbon available for the L-Lys route into L-Ile production. The L-Ile yield of the constructed strain was improved by 50-fold with a resultant level of 2.5 g/L, along with a reduction in L-Lys yield by 69.1% [207]. A TD-overexpression mutant (V140M-F383A) displayed higher specificity activity and complete resistance to L-Ile. The recombinant *C. glutamicum* strain ATCC14067/*ilvA*^V140M-F383A^ increased L-Ile yield by 55.3% with a titer of 0.73 g/L [207]. In addition, one copy of feedback-resistant HD (G378E) and the TD (V323A) genes *hom^fbr^* and *ilvA^fbr^* were introduced into the L-Lys-producing *C. glutamicum* MH20-22B by homologous recombination. Further expression of *ilvA^fbr^* from a plasmid in the strain improved L-Ile synthesis to 138 mM with a yield of 0.11 mol/mol and a productivity of 0.28 mmol/g per hour by fed-batch cultivation [208]. DR17/pECM3::*ilvA*(H278R-L351S) was derived from MH20-22B, in which three copies of *hom^fbr^* encoding feedback-resistant HD were introduced, and *ilvA^fbr^* encoding feedback-resistant TD was overexpressed from a multicopy plasmid. The strain produced 96 mM L-Ile at a yield of 0.14 g/g glucose through batch culture [8]. Although high-level expression of *hom^fbr^*, *thrB*, and *ilvA^fbr^* (V328A) in MH20-22B, an engineered strain of *C. glutamicum* SM13, produced only 60 mM L-Ile extracellularly at a yield of 0.22 g/g glucose, the intracellular L-Ile rose to 110 mM [209]. Moreover, co-amplification of the feedback-tolerant TD (F383A) gene *ilvA^fbr^* and AHAS (P176S, D426E, and L575W) gene *ilvBN^fbr^* in the L-Ile producer *C. glutamicum* JHI3-156 improved the L-Ile titer to 30.7 g/L after 72 h in fed-batch cultivation [210]. The feedback-resistant TD gene *ilvA^fbr^*, active AHASII gene *ilvGM*, DHAD gene *ilvD*, and AT gene *ilvE* were also introduced by a plasmid into the engineered L-Thr producer *E. coli* TDH6 (RSF1010*thrA^fbr^BC*). The constructed strain synthesized 10.2 g/L L-Ile after 24 h at a yield of 0.26 g/g glucose [211]. Upon further amplification of the feedback-resistant AKIII gene *lysC^fbr^* in the strain, production improved to 12.3 g/L [17]. In addition, cysteine synthase A was overexpressed in an L-Ile producer *C. glutamicum* IWJ001 to upregulate the genes *aspC*, *lysC*, *hom*, *thrB*, *ilvA*, and *ilvBN* involved in L-Ile biosynthesis. The recombinant strain increased L-Ile yield by 23.5% at a titer of 25.33 g/L, with productivity of 0.352 g/L per hour [212].

Competing branches represent another engineering target for L-Ile production. The genes *ddh* and *lysE* were first disrupted in *C. glutamicum* ATCC13869 to reduce the production of the by-product L-Lys. Sequentially, a plasmid harboring the key L-Ile pathway genes *lysC^fbr^*, *hom^fbr^*, *thrB*, and *ilvA^fbr^* was transformed into the mutant. The engineered strain generated 3.75 g/L L-Ile in batch cultivation [197]. A plasmid-free L-Thr hyper-producing strain, K2P55, was derived from the undirected mutagenesis L-Lys producer *C. glutamicum* MH20-22B. In K2P55, the feedback control of HD and TD was removed by introducing point mutations *hom* (G378E) and *ilvA* (V323A) into the chromosome, and the expression levels of *hom^fbr^* and *ilvA^fbr^* were enhanced by mutating the promoter. The expression level of the *dapA* gene was reduced by introducing the P*dapA*-C13 promoter mutation to inhibit the L-Lys formation. *C. glutamicum* K2P55 could produce 109 mM L-Ile with a yield of 0.19 mol/mol [86]. In turn, the disruption of *alaT* encoding alanine aminotransferase in the L-Ile producer *C. glutamicum* YILW blocked alanine formation and increased precursor PYR availability. The expression of the gene *thrABC* from the industrial L-Thr producer *E. coli* TRFC in the mutant to increase the precursor 2-ketobutyrate yielded a final strain that generated L-Ile at 14.2 g/L [213]. Zhang et al. performed a comprehensive transcriptomic analysis, demonstrating that disruption of the L-Thr metabolic pathway led to significant perturbations in genes related to L-Ile synthesis pathways. Based on this insight, they successfully constructed a chassis strain that exhibited an enhanced L-Ile yield of 7.48 g/L [87].

### 8.2. Engineering to Enhance NADPH Supply for L-Ile Production

Apart from the biosynthesis pathway, NADPH provision also comprises a key factor for L-Ile generation. Overexpressing the NAD kinase gene *ppnk* in JHI3-156 successfully increased L-Ile yield by 36.1% at 0.049 g/g glucose at a titer of 2.89 g/L [214]. Similarly, the PPP 6-phosphogluconolactonase gene *pgl* and 6PGD gene *gnd*, which are involved in NADPH synthesis, were amplified in the L-Ile industrial producer *C. glutamicum* IWJ-001, with the recombinant strain producing L-Ile at a yield of 0.15 g/g glucose. Further expression of *fbp* encoding FBP enhanced L-Ile productivity to 0.11 g/L per hour by shake flask cultivation by redirecting the carbon flux to PPP [215]. Co-expression of *ppnk* and the NADP^+^-dependent G6PD gene *zwf* significantly improved NADPH provision, increasing L-Ile generation by 85.9% at a level of 4.1 g/L [84]. In addition, the NAD kinase Ppnk was amplified in combination with TD and AHAS in the L-Ile producer *C. glutamicum* IWJ001 to increase NADPH and L-Ile carbon flux. The developed strain markedly enhanced L-Ile formation to a titer of 32.3 g/L at a yield of 0.12 g/g glucose using fed-batch fermentation, but only less than 21% of the theoretical yield [85]. Importantly, this represents the highest publicly reported titer of L-Ile achieved through microbial fermentation.

### 8.3. Engineering to Improve Secretion for L-Ile Production

Beyond engineering L-Ile biosynthesis, competitive branches, and NADPH supply, the secretion system has become an important target for further increasing L-Ile production. Amplifying the regulatory factor Lrp and the transporter BrnFE in JHI3-156 increased L-Ile synthesis by 63% by shake flask culture, with the L-Ile specific yield being enhanced by 72% in fed-batch fermentation [216]. Deleting the L-Ile uptake carrier BrnQ gene *brnQ* from the chromosome of the L-Ile producer *C. glutamicum* YILW reduced the L-Ile uptake rate. The *brnFE* gene was also overexpressed in this strain. L-Ile generation by the strain achieved 29 g/L within 60 h at a yield of 0.24 g/g glucose using fed-batch fermentation [88].

## 9. Conclusions and Perspective

**Figure 4:**
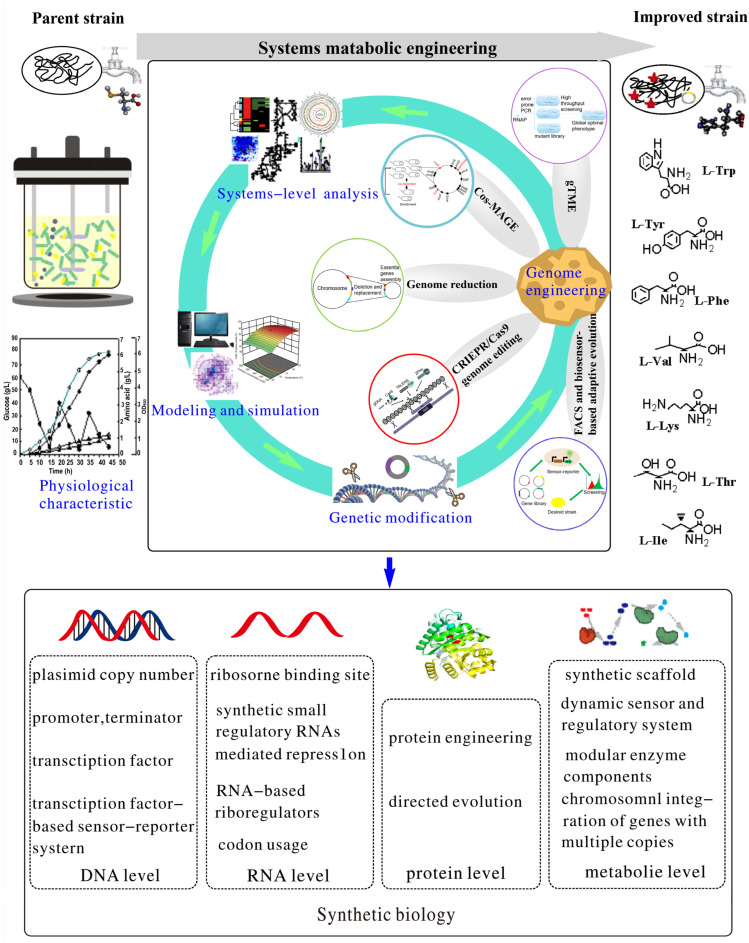
Systems metabolic engineering of microorganisms for amino acid production based on systems biology, synthetic biology, and genome engineering.

The POP node as the switch point for carbon flux is widely distributed in bacteria. A series of amino acids from the central metabolic node can be generated based on a specific branched point and corresponding biosynthesis pathway. To enhance the productivity of the products, enzyme and metabolic engineering strategies have been systematically investigated to deregulate feedback inhibition, improve the catalytic performance, block competitive pathways, enhance amino acid exportation, modify the cofactor generation, and strengthen the provision of PEP, PYR, and OAA (Figure 4). By deregulating feedback inhibition and improving the catalytic performance, we can directly guide the synthesis pathway towards the desired product and precisely modulate the metabolic flow, ultimately resulting in exceptional outcomes. However, a profound understanding of the gene regulatory network is crucial, as it may potentially influence other metabolic pathways and trigger unintended side effects. Blocking competitive pathways can enhance the purity of the target product, minimizing resource wastage, but it could also disrupt the overall metabolic balance of the cell. Transport engineering techniques can boost the substrate’s entry into the cell or facilitate the export of amino acid products, yet this may increase cellular energy expenditure and hinder growth. Modifying the cofactor generation involves altering cofactor production pathways or enhancing their regeneration efficiency. However, the intricate cofactor network could potentially trigger a chain reaction. Optimizing carbon flow through strengthening the provision of PEP, PYR, and OAA is a strategy for augmenting metabolic flux, leading to an increase in target product titer. Nevertheless, this could potentially impact cell growth and product diversity. As metabolic engineering progresses, these metabolic engineering strategies have become fundamental to the initial construction of target products. The strategies work synergistically, complementing each other, ultimately achieving significant results in the production of POP-AAs.

Despite an array of significant developments being achieved, three major challenges remain and restrict the efficient production of the target amino acids. The first enzyme that branches out of the central metabolism has a lower affinity to PEP, PYR, and OAA than PYK, PDHC, and CS. Meanwhile, only the branches towards the generation of DAHP, ACL, and ASP serving PEP, PYR, and OAA as substrates are satisfied for target amino acid production. Therefore, inadequate substrate affinity and enzyme activity of DAHPS, AHAS, and AAT constrain product production. To address the challenge, computer-based enzyme engineering has been successfully implemented and will be continued, encompassing screenings for novel branches of an enzyme with enhanced reaction efficiency across species, rational design of amino acid synthesis pathway enzymes to boost activity, mutagenesis of rate-limiting enzymes prone to substrate inhibition, and the utilization of protein engineering to modify coenzyme specificity for optimized cofactor utilization.

On the other hand, POP-AAs are partially coupled with cell growth products whose levels are dramatically affected by the metabolic flux of the refilling node, TCA cycle, and target amino acid biosynthesis pathways. In this respect, it is essential to supply appropriate precursors for PEP, PYR, and OAA depending on the real-time demand of the organisms for the biosynthesis of target compounds. Nevertheless, the concentrations of PEP, PYR, and OAA strongly fluctuate under the different growth conditions and stages, which creates challenges for achieving a balance between the target product and cell growth. Dynamic regulation strategies will be vital to enhancing target amino acid production. Specifically, cell growth and synthesis of products can be decoupled by the CRISPR-dCpf1 system [217,218]. Compared to Cas9, Cpf1 (the class 2 type V-A CRISPR effector) not only possesses the activity of both DNase and RNase, enabling it to process crRNA arrays into mature crRNAs and offering a viable alternative for genetic editing [219], but also has a significantly shorter direct repeat sequence (~20 nt) compared to the handle sequence of Cas9 crRNA (~60 nt) [220,221]. This shorter sequence makes the synthesis and assembly of Cpf1 crRNA arrays more cost-effective and simpler. When the inducer IPTG was supplied in the early-logarithmic growth phase, the CRISPR-dCpf1 system was turned on, causing repression of the expression of the *pyk*, *ppc*, *aceE*, *pyc*, *pck*, and *gltA* genes by dCpf1 and crRNA binding to the RBS of the target gene. The expression of critical genes in the pathway was enhanced for target compound biosynthesis simultaneously using the dCpf1, crRNA, and RNAP adhering to the promoter region of the target gene [222]. Therefore, the metabolic flux of the TCA cycle and anaplerosis was limited. Moreover, the metabolized glucose was channeled toward the target amino acid pathway.

Cofactor engineering has been developed to promote POP-AA production [57,174]. Microorganic cofactor redox balance could be maintained by regulating endogenous cofactor systems and installing heterologous cofactor regeneration systems. The redox metabolism of the TCA cycle under aerobic conditions generates sufficient reduced cofactors (NADPH, NADH, and ATP), which are required for both microorganism growth and the synthetic pathway of target amino acids. However, in POP-AA fermentation, one-third of the carbon-based materials are oxidized into CO_2_ through the redox metabolism of the TCA cycle. Therefore, a TCA-cycle-independent cofactor regeneration system and CO_2_ reassimilation are desirable for target amino acid biosynthesis. Extracellular reducing power from electrons must be taken up for cofactor regeneration and metabolic utilization of CO_2_. Electricity generated from regenerative energy is possibly the most sustainable and cost-competitive form. Nevertheless, the molecular mechanism of electric energy transfer in microorganisms still needs to be discovered. The electrochemical process determined so far for electro-biosynthesis is far from practical utilization efficiency. *Cyanobacteria* can directly use CO_2_ and be genetically engineered to generate valuable chemicals [223,224]. Meanwhile, CO_2_ fixation has also been developed in *E. coli* [225,226,227,228,229,230]. Researchers are attempting to engineer *E. coli* to grow photoautotrophically by introducing heterologous C_1_ assimilation pathways, i.e., the Calvin–Benson cycle, the Wood–Ljungdahl pathway, or the dicarboxylate/4-hydroxybutyrate pathway. Based on this, a promising integrated photosynthetic system for target amino acid generation might be explored in *E. coli*.

In addition, maintaining fitness and robustness and increasing the environmental tolerance of amino acid producer strains is critical for industrial processing. Genome reduction through the precise disruption of dispensable genes and other sequences containing all known recombinogenic foreign DNA and cryptic virulence genes may help enhance cell stability and streamline metabolism without physiological compromise. Global transcription machinery engineering and adaptive laboratory evolution may also aid metabolic engineering in improving tolerance towards harsh industrial environments (Figure 4).

Ultimately, achieving high titer, yield, and productivity is crucial for amino acid products to gain market competitiveness. Despite significant efforts in constructing POP-AA-producing strains via metabolic engineering strategies, developing microbial cell factories with industrial-scale competitiveness for POP-AA production remains a significant challenge. Maximizing production hinges on several critical aspects: broadening the substrate spectrum to include novel and inexpensive carbon and energy sources, optimizing endogenous substrate utilization pathways, balancing cell growth with amino acid synthesis, maintaining cofactor redox balance, and efficiently transporting intracellular target amino acids. The delicate balance between biomass accumulation and production efficiency represents the core challenge and bottleneck in effective POP-AA synthesis. The integration of rational metabolic engineering, coupled with the application of novel synthetic biology tools, offers promising solutions to this issue. Dynamic regulation remains a viable metabolic modification strategy for significantly enhancing the biosynthesis of POP-AAs [231].

In the future, metabolic engineering strategies encompassing dynamic regulation, high-throughput screening, and omics analysis will remain crucial in enhancing the efficient production of POP-AAs and will be further optimized to address the aforementioned bottlenecks. The evolution from random strain mutations to the integration of rational metabolic engineering and the growing utilization of novel metabolic engineering techniques have propelled the progress of microbial POP-AA production. With the ongoing advancements in systems metabolic engineering, we expect to adopt more advanced and precise strategies to construct high-performing industrial strains. The metabolic engineering approach to POP-AA production provides valuable insights, indicating that the biosynthesis of other AAs or derivatives will become more efficient, accurate, and cost-effective.

## Figures and Tables

**Table 1:** Representative metabolic engineering strategies for strain development for the production of glycolysis-derived amino acids.

Strain	Engineering Strategy	Culture Method	Titer (g/L)	Yield (g/g)	Productivity (g/L/h)	References
L-tryptophan						
*C. glutamicum* KY9218 (pKW9901)	↑*aroG^fbr^*, ↑*trpE^fbr^D^fbr^CBA*, ↑*serA*	Fed-batch	50	0.23	0.63	[25]
*C. glutamicum* KY9218 (pIK9960)	↑*tktA*, ↑*aroG^fbr^*, ↑*trpE^fbr^D^fbr^BA*, ↑*serA*	Fed-batch	58	ns	ns	[26]
*E. coli* FB-04 (pSV03)	↑*aroF^fbr^*, ↑*trpE^fbr^D*, *ΔtrpR*, *ΔtnaA*, *ΔpheA*, *ΔtyrA*	Batch culture	13.1	0.1	ns	[27]
*E. coli* TRTH1013	*Δtna*, *ΔtrpR*, ↑*tktA*, ↑*ppsA*, ↑*aroG^fbr^*, ↑*trpE^fbr^DCBA*, ↑*serA*	Fed-batch	40.2	0.17	ns	[28]
*E. coli* T13	*ycjv*::P*_trc_-aroEK*, *mbhA*::P*_trc_-glnA*^L159I,E304A^, *yeeP*::P*_serB_-serB*, P*_serC_-serC*, *ΔTnaB*	Fed-batch	53.65	0.238	ns	[29]
*E. coli* TRTH03	*ΔtnaA*, *Δmtr*, *ΔpykA*, *Δppc*, ↑*trpE*^fbr^, ↑*aroG*^fbr^, ↑*pck*, ↑*citT*, ↑*acnBA*, ↑*icd*, ↑*pyc*	Fed-batch	49	0.19	ns	[30]
*E. coli* SX11	*yghX*::P*_trc_-xfpk, ptsG*::P*_M1-12_-glf*, *ycjv*::P*_M1-12_-glk*, *ΔpykF*, *ylbE*::P*pck-pck, mbhA*::P*_trc_-pyc*^P458S^	Fed-batch	41.7	0.227	1.04	[31]
*E. coli* GPT1017	*Δmtr*, *ΔtnaB*, *ΔaroP*, ↑*tktA*, ↑*aroG^fbr^*, ↑*trpE^fbr^*	Fed-batch	16.3	ns	0.25	[32]
*E. coli* TRTH03	P*ara-acs*::*araB*; P*ara*-*aceB*-*mdh*-*pck*::*yghx*	Fed-batch	54.6	ns	ns	[33]
*E. coli* MG1655-Y	*Δpta*, *Δfmtr*, ↑*yddG*, ↑*tktA*, ↑*ppsA*	Fed-batch	48.7	0.22	1.39	[34]
*E. coli* Trp30	*ΔtrpR*, *ΔtnaA*, P2-*trpE^S40F^DCBA*, *ΔackA*, *Δpta, ΔpoxB*	Fed-batch	42.5	0.178	0.89	[35]
L-tyrosine						
*E. coli* T2	↑*tktA*, ↑*pps*, ↑*aroG^fbr^*, ↑*tyrA^fbr^*, *ΔftyrR*	Fed-batch	9.7	0.1	ns	[36]
*E. coli* DPD4193	↑*aroG^fbr^*, *ΔpheAL*, ↑*tyrA^fbr^*, ↑*cscB*, ↑*cscK*, ↑*cscA*	Fed-batch	55	ns	1.22	[37,38]
*E. coli* JH7	↑*P_L_lacO*-1-*phhA*-*phhB*-*folM*-*T1*, ↑*P_L_lacO*-1-*mtrA*-*folX*-*T1*-*P_L_lacO*-1-*aroL*-*ppsA*-*tktA*-*aroG^fbr^*	Shake flask	0.401	ns	ns	[39]
*E. coli* W3110	*ΔtyrR*, *ΔtyrA*, *ΔfpheA*, ↑*aroG^fbr^*, ↑*tyrA^fbr^*, ↑*aroL*	Shake flask	6.3	0.16	ns	[40]
*E. coli* BL21(DE3)	↑P*tac-aroG*^fbr^-*aroL*, P*trc-tyrC*, *ΔtyrP*	Fed-batch	43.14	0.11	ns	[41]
*C. glutamicum* KY10865 (pKY1)	↑*aroG^fbr^*, ↑*pheA^CM^*	Fed-batch	26	ns	ns	[42]
*E. coli* HGD (M9)	*ΔtyrR*, *ΔpheA*, *ΔtrpE*, *ΔaroP*, *ΔtyrP*, *dadX*-*cvrA*::P*_j231119_*-*yddG*, *tyrP*::P*_j231119_*-*tktA*, *trpE*::P*_j231119_*-*ppsA*, *ykgH*-*betA*::P*_j231119_*-*udhA*, *yeeJ*-*yeeL*::P*_j231119_*-*pntAB*, *ΔpoxB*, pAP with *aroG^fbr^*, *tyrA^fbr^*, *fpk*, *pta*, p15A ori with P_R_P_L_	Fed-batch	92.5	0.266	ns	[43]
*E. coli* S17-1	↓*tyrR*, ↓*csrA*, ↑*pgi*, ↑*ppc*	Fed-batch	21.9	ns	ns	[44]
L-phenylalanine						
*E. coli* BR-42 (pAP-B03)	↑*pheA^fbr^*, ↑*aroF*	Two-stage process	57.63	ns	1.15	[45]
*E. coli* xllp1	↑P*_tyrP_*-*aroK*	Fed-batch	61.3	0.22	1.27	[46]
*E. coli* DV269 (TyrA-LAA)	↓*tyrA*	Batch culture	7.2	0.14	0.3	[47]
*E. Coli* AJ12741 (pHYGG)	↑*yddG*	Shake flask	6.4	0.16	ns	[48]
*B. flavum* 311 (pJN5)	↑*ppsA*, ↑*pckA*, ↑*aroG*, ↑*pheA*, ↑*tyrB*	Shake flask	5.39	ns	ns	[49]
*E. coli* PB12-ev2	↑*aroG^fbr^*, ↑*pheA^ev2^*, ↑*tktA*, *ΔptsHI*, *galP*	Shake flask	ns	0.33	40 ^a^	[50]
*C. glutamicum ΔptsI*::*iolT2*-*ppgkΔaroP* *ΔaceEΔldh* (pSUTL, pSDTL)	↑*aroF^fbr^*, ↑*aroE*, ↑*ppsA*, ↑*tktA*, ↑*pheA^fbr^*, ↑*aroA*, ↑*tyrB*, ↑*aroL*, ↑*iolT2*-*ppgk*, *ΔfpstI*, *ΔfaroP*, *ΔfldhA*, *ΔfaceE*	Fed-batch	15.76	ns	0.197	[51]
*E. coli* Xllp08	*ΔptsH*, ↑*galp*, ↑*glk*, *TyrR*T495I, ↑*aroD*^fbr^	Fed-batch	72.9	0.26	1.4	[52]
*E. coli* WF123456+*fis*	↑*aroKB*, ↑*aroG^fbr^*, ↑*tktA*, *ΔtyrR*, ↑*fis*	Shake flask	0.9	0.083	0.032	[53]
*E. coli* PHE05	↑*aroK1*, ↑*aroL1*, ↑*pheA1*, ↑*aroA*, ↑*aroC*, ↑*tyrB*, adaptive evolution	Fed-batch	80.48	0.27	1.68	[54]

ns, not specified. ^a^ mg/g-dcw/h.

**Table 2:** Representative metabolic engineering strategies for strain development for the production of Krebs cycle-derived amino acids.

Strain	Engineering Strategy	Culture Method	Titer (g/L)	Yield (g/g)	Productivity (g/L/h)	References
L-valine						
*C. glutamicum ΔilvAΔpanB ilvN*M13 (pECKAilvBNC)	↑*ilvBNC*, *ilvN^M13^*, *ΔilvA*, *ΔpanB*	Batch culture	15.2	0.38	0.32	[55]
*C. glutamicum aceE* ^A16^*ΔpqoΔppc* (pJC4ilvBNCE)	↓*aceE*, ↑*ilvBNCE*, *Δppc*, *Δpqo*	Fed-batch	86.5	0.23	1.6	[56]
*C. glutamicum ΔLDH* BN^GE^C^TM^DLD	↑*ilvBN^fbr^*, ↑*ilvC^TM^*, ↑*ilvD*, ↑*LeuDH*	Anaerobic, fed-batch	227.3	0.41	ns	[57]
*E. coli* W *ΔilvA* (pTrc184ygaZHlrp, pKBRilvBN^fbr^CED)	*ΔilvA*, ↑*ilvBN^fbr^CED*, ↑*lrp*, ↑*ygaZH*	Fed-batch	60.7	ns	2.06	[58]
*C. glutamicumΔppcΔpyc*ICD^G407S^	*Δppc*, *Δpyc*, ICD^G407S^, *ilvBNCE*	Shake flask	8.9	0.22	ns	[59]
*Bacillus subtilis* AW015-5	↑*ilvBH^fbr^C*, ↑*ilvD*, ↑*ybge*,↑ *ywaA*, ↓*bcd*, ↓*pdhA*, *ΔleuA*, *ΔilvA*,	Shake flask	4.61	ns	ns	[60]
L-lysine						
*E. coli* LATR11 (pWG-*DC^SM^A^SM^BH_c.g_LP*)	↑*ppc*, ↑*lysC*^T344M^, ↑*asd*, ↑*dapA*^H56K^, ↑*dapB*, ↑*lysA*, ↑*ddh*	Fed-batch	125.6	0.59	3.14	[61]
*C. glutamicum* LYS-12	↑*ddh*, ↑*lysA*, ↑*lysC*^T311I^, ↑*dapB*, *hom*^V59A^, *Δpck*, ↑*pyc*^P458S^, ↓*icd*, ↑*fbp*, ↑*zwf*-*opcA*-*pgl*-*gnd*	Fed-batch	120	0.55	4	[62]
*C. glutamicum* DRL2	*Δpyk*, *ppc*_D299N_, *gltA*_S252C_	Shake flask	15.7	ns	0.56	[63]
*C. glutamicum* JL-6 9P*_tac-M_ gdh*	*ppc*::*pck*, *pyc*::*odx, Δ*P1*_gltA_*, P*_tac-M_*::P*_gdh_*	Fed-batch	181.5	ns	3.78	[64]
*C. glutamicum* Lys9	*ΔaceE*, *pyc*^C1372T/G1A^, *ΔalaT*, *ΔavtA*, *ΔldhA*, *Δpck*, *Δmdh*, ↑*lysC*^C932T^, ↑*pntAB*	Fed-batch	76.8	0.42	2.69	[65]
*C. glutamicum* ATCC13032 Psod^fbp^-zwf243	↑*zwf*^A243T^, ↑*fbp*	Shake flask	ns	0.13	ns	[66]
*C. glutamicum* RE2A^iol^/pCAK311	*gapN*::*gapA*, *rho*^R696C^, *ΔgapB*, *gapA*::*iolT1*	Shake flask	9.3	ns	0.29	[67]
*C. glutamicum* JL-6	*Ec*-*dapB*^C115G,G116C^::*dapB*	Fed-batch	117.3	0.44	2.93	[68]
*C. glutamicum* GRLys1	↑*iolT2*,↑*glcK*, *ΔsugR*, *ΔldhA*	Shake flask	4.81	ns	0.22	[69]
*C. glutamicum* ZL-92	↑*ptsIH*, *ΔsugR*, ↑*iolT1*, ↑*iolT2*, ↑*ppgK*	Fed-batch	201.6	0.65	5.04	[70]
*C. glutamicum Δhom*::HPA	↑*amyA*, ↑*pgsA*	Batch culture	6.0	0.19	ns	[71]
*E. coli* QD01 Δ*tRNA L2*	establishment of artificial rare cryptosystem for mutating and screening	Shake flask	14.8	ns	ns	[72]
L-threonine						
*E. coli* TH-103Z	artificial construction of polyploid *Escherichia coli*	Fed-batch	160.3	ns	ns	[73]
*C. glutamicum* R102*ΔmetXΔdapA*(pEX-Box)	*ΔmetX*, *ΔdapA*, ↑*hom*-*thrB*, ↑*thrE*	Shake flask	3.5	0.045	0.049	[74]
*C. glutamicum D*	*ΔilvA*, *ΔmetX*, *ΔtdcB*, ↑*lys^fbr^*, ↑*hom^fbr^*M, ↑*asd*, ↑*thrB*, ↑*thrC*	Shake flask	12	ns	ns	[75]
*E. coli* TWF044	*ΔfadR*, *ΔfabR*, *ΔlacI*, *fadBA*::*lacZ*, P*_tac_-_trc_*:P*acs*, P*_tac_*-*thrA^fbr^BC*-*rhtC*::*lacA*, P*_tac_*-*ppnk*-*aspC*-*ppc*::*lacZ*	Fed-batch	103.9	0.72	2.16	[76]
*E. coli* WMZ016/pFW01-thrA*BC-*rhtC*	*Δcrr*, *ΔiclR*, P*trc*::P*gltA*, ↑*thrA^fbr^BC*-*rhtC*	Shake flask	18.0	0.35	ns	[77]
*E. coli* THRD *ΔpfkBΔpykF*	↑*pfkB*, ↑*pykF*	Fed-batch	111.4	0.37	3.98	[78]
*E. coli* TWF083	*RBS_thrL_*-*thrL*-*RBS_thrA_*::*iclR*, P*_cysH_*-*RBS_s7_*-*aspC*::*lacI*, ↓*arcA*, *fadR*, *cpxR*, *gadE*, *pykF* dynamic regulated by *thrR*	Fed-batch	116.6	0.49	2.43	[79]
*E. coli* MG422 *rhtA23* (pVIC40)	↑*thrA^fbr^BC*, ↑*rhtA*	Shake flask	36.3	0.73	0.79	[80]
*E. coli* TWF113/pFT24rpa1	*ΔpoxB*, *ΔpflB*, *ΔldhA*, *ΔadhE*, *ΔtdcC*, *ΔavtA*, *ΔalaA*, *ΔalaC*, *cI^ts^*-*P_R_*-*P_L_rhtC-pyc*, *tetR*-*P_LtetO-1_-alaA*	Fed-batch	ns	124.03%	ns	[81]
*E. coli* (*P*_2.1_*-2901* Δ*ptsG*)	dynamical regulation of the expression of the *thrABC* operon, *ΔptsG*	Shake flask	40.06	ns	ns	[82]
*E. coli* MDS205	↑*thrA*^fbr^*BC*, *Δtdh*, *rhtA23*::*tdcC*, *rhtA23*::*sstT*	Batch culture	40.1	0.4	1.34	[83]
L-isoleucine						
*E. coli* TLCD	↑*ilvGMEDA^fbr^*, ↑*thrA^fbr^BC*, ↑*lysC^fbr^*	Shake flask	12.3	0.23	0.51	[17]
*C. glutamicum* JHI3-156	↑*ppnk*, ↑*zwf*	Shake flask	4.1	0.029	0.057	[84]
*C. glutamicum* IWJ001	↑*ilvBN*1, ↑*ilvA*1, ↑*ppnk*1	Fed-batch	32.3	ns	ns	[85]
*C. glutamicum* K2P55	↑*hom^fbr^*, ↑*ilvA^fbr^*, ↓*dapA*	Fed-batch	14.3	0.14	0.18	[86]
*E. coli* NXU102	*ΔleuA*/Met^−^*+* Lys^−^/*Δtdh*/*ΔltaE*/*ΔyiaY*	Shake flask	7.48	ns	ns	[87]
*C. glutamicum* YILW*ΔbrnQ*pXMJ19*brnFE*	*ΔbrnQ*, ↑*brnFE*	Fed-batch	29	0.24	0.48	[88]

ns, not specified.

## Data Availability

Date sharing is not applicable.
